# Chronic sun exposure-related fusion oncogenes EGFR-PPARGC1A in cutaneous squamous cell carcinoma

**DOI:** 10.1038/s41598-017-12836-z

**Published:** 2017-10-04

**Authors:** Sho Egashira, Masatoshi Jinnin, Manami Ajino, Naoki Shimozono, Sayo Okamoto, Yukino Tasaki, Ayaka Hirano, Maho Ide, Ikko Kajihara, Jun Aoi, Miho Harada, Toshikatsu Igata, Shinichi Masuguchi, Satoshi Fukushima, Hironobu Ihn

**Affiliations:** Department of Dermatology and Plastic Surgery, Faculty of Life Sciences, Kumamoto University, 1-1-1 Honjo, Chuo-ku, Kumamoto 860-8556 Japan

## Abstract

Cutaneous squamous cell carcinoma (cSCC) differs from SCC of other organs in its strong association with chronic sun exposure. However, the specific driver mutations in cSCC remain unknown. Fusion genes in established cSCC cell lines (A431 and DJM-1) were predicted by transcriptome sequence, and validated by Sanger sequence, fluorescence *in situ* hybridization and G-banding. By transcriptome sequencing, we identified fusion gene EGFR-PPARGC1A in A431, which were expressed in 31 of 102 cSCCs. The lesions harboring the fusion gene tended to be located in sun-exposed areas. *In vivo* cutaneous implantation of EGFR-PPARGC1A-expressing NIH3T3 induced tumors resembling human cSCC, indicating its potent tumorigenicity. NIH3T3 transfected with EGFR-PPARGC1A as well as A431 showed increased cell proliferation activity. With regard to underlying mechanism, EGFR-PPARGC1A protein causes constitutive tyrosine phosphorylation, and induces the phosphorylation of wild-type full-length epidermal growth factor receptor (EGFR) by dimerization. Conversely, the RNAi-mediated attenuation of EGFR or CRISPR/Cas9-mediated knockdown of the fusion gene in A431 led to a decrease in the cell number, and may have therapeutic value. Our findings advance the knowledge concerning genetic causes of cSCC and the function of EGFR, with potential implications for new diagnostic and therapeutic approaches.

## Introduction

Cutaneous squamous cell carcinoma (cSCC), which originates from the keratinocytes of the epidermis in elderly patients, is one of the most common types of skin cancer. Although SCC can be seen in many other internal organs, including the esophagus, lungs, and urinary bladder, cSCC is characterized by its strong association with chronic and cumulative sun exposure.

Most of cSCCs are considered low risk and can be treated by local excision. However, when they progress, their potential to recur and metastasize leads to a poor prognosis and is associated with significant mortality. Although an early diagnosis is important, some atypical or well-differentiated cases are difficult to diagnose clinically or histopathologically. At present, the serum SCC antigen level is the only available tumor marker. Unfortunately, this marker only becomes elevated at the late stage^1^. Furthermore, although radiotherapy and chemotherapy have been utilized in the treatment of advanced cSCC, the tumor is sometimes resistant to such standard treatments. Clarifying the pathogenesis of this tumor may be helpful for the development of novel biomarkers and new therapeutic strategies.

We previously reported that various cytokines and microRNAs are involved in the pathogenesis of cSCC^2–5^. On the other hand, other publications have reported several genetic abnormalities, including *TP53* mutations, in cSCC^6^. However, there may be more specific driver mutations in this tumor. To date, chromosomal translocation with corresponding fusion genes has been widely identified in various tumors, especially mesenchymal neoplasms and sarcomas. Among skin tumors, dermatofibrosarcoma protuberans is associated with t(17;22) translocation. The t(17;22) translocation causes *COL1A1-PDGFB* rearrangement, and COL1A1-PDGF fusion protein stimulates PDGF receptor, leading to malignant transformation^7^. Because *COL1A1-PDGFB* is highly specific to dermatofibrosarcoma protuberans, the detection is clinically helpful for the diagnosis. In addition, imatinib has been found to have anti-tumor effect through its inhibition of fusion gene-induced PDGF receptor activation. Accordingly, the identification of fusion genes in malignant tumors is important for the development of novel treatments. However, no specific fusion genes have been identified in cSCC.

In the present study, we describe fusion gene *EGFR-PPARGC1A*, which was found in 31 of 102 cSCC patients. We analyzed the clinical significance, and proved that the fusion play causal roles in tumor development *in vitro* and *in vivo*.

## Result

### The identification of cSCC-specific fusion genes in A431

High-quality RNAs were isolated from NHEKs or two cSCC cell lines (A431 and DJM-1), and were analyzed by paired-end transcriptome sequencing on Illumina HiSeq2000 platform: among all of the reads, 36,796,013 (90.48%) for NHEKs, 36,748,245 (89.80%) for A431, and 39,474,844 (90.38%) for DJM-1 were mapped to the reference genome (GRCh37/hg19).

Initially, for the analysis of the differential gene expression between A431 and NHEKs, fragments per kilobase per million map reads (FPKM) values were calculated, and the statistical significance was evaluated using Cuffdiff (Supplementary Fig. S1A). As shown in Supplementary Table S1, when the cut-off value was set at 2^8^-fold-change with false discovery rate (FDR) of <0.01, 16 or 54 genes were estimated to be substantially up- or down-regulated in A431 in comparison to NHEKs, respectively. Several of these changes in A431 that were found in the present study were compatible with previous reports: For example, *MAGEA4*, *MMP13*, and *SPP1*, which have been reported to be overexpressed in cSCC cells *in vivo* and *in vitro* ^8–10^, were also up-regulated in A431 in our transcriptome analysis. However, the cluster analysis did not indicate a specific signature of signal pathways or cellular activities (Supplementary Fig. S1B).

Thus, we next examined the presence of A431-specific point mutations in seven genes related to SCC of other organs and 36 oncogenes. In addition to several neutral polymorphisms and amino acid substitutions that represent common variations, 13 single nucleotide variations were detected in A431 when compared to NHEKs and DJM-1, as well as the reference genome in bioinformatic analysis (Supplementary Table S2). Among these, *TP5*3 R273H point mutation had already been described in A431^11^.

**Figure 1:**
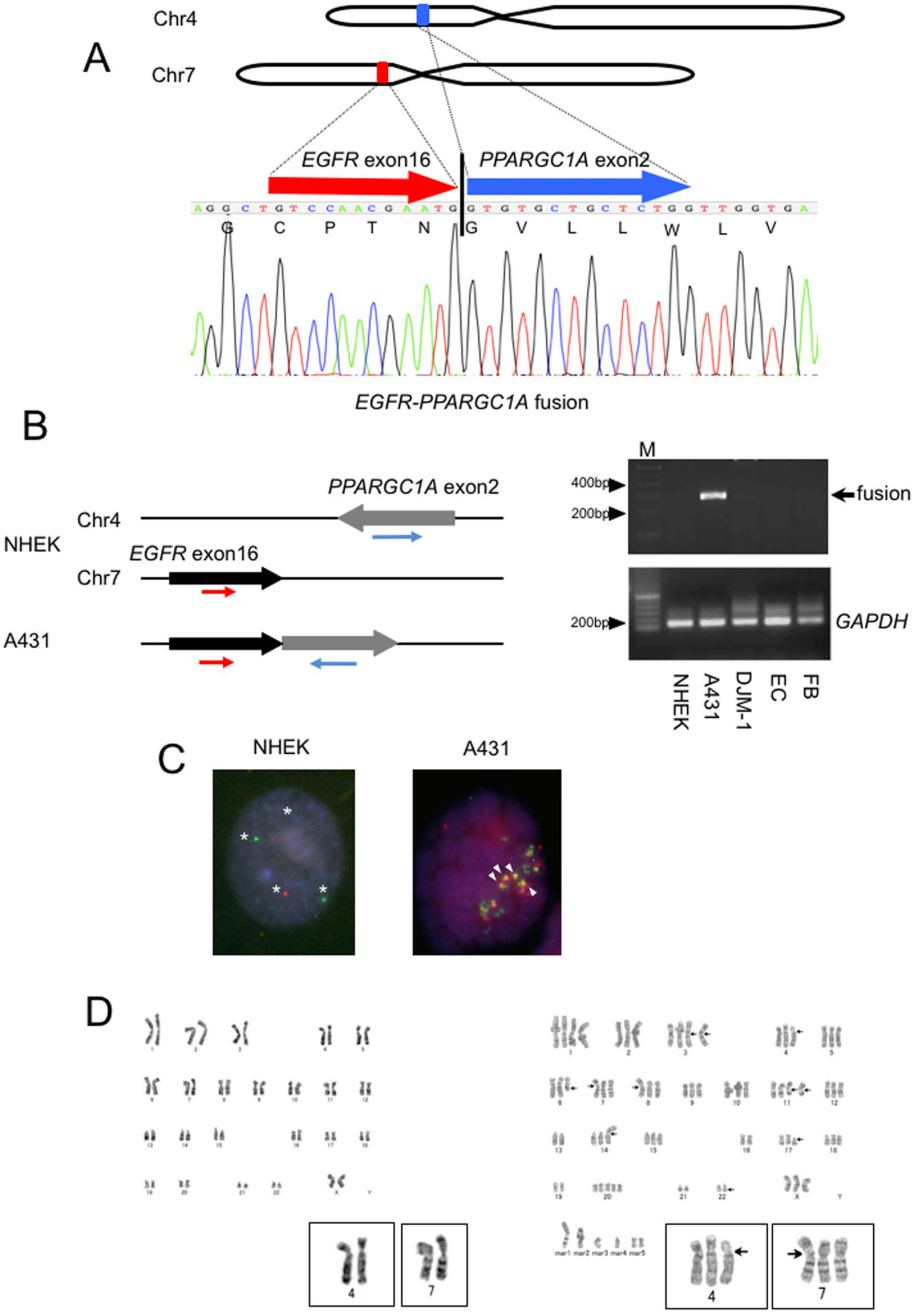
The identification of a novel *EGFR-PPARGC1A* fusion in A431. (**A**) (Upper panel) A schematic diagram showing gene structures of *EGFR* (red) and *PPARGC1A* (blue) in relation to the formation of the *EGFR-PPARGC1A* fusion transcript. (Lower panel) The partial sequence of the *EGFR-PPARGC1A* fusion transcript at the break point, along with the predicted amino acid sequence. The transcript is a fusion of *EGFR* exon 16 to *PPARGC1A* exon 2. (**B**) (Left panel) The RT-PCR-based detection of the *EGFR-PPARGC1A* fusion. The presence of the *EGFR-PPARGC1A* fusion transcript was verified by RT-PCR using the *EGFR* forward (red) and *PPARGC1A* reverse primers (blue). The fusion gene-specific primers were designed to not amplify the wild-type full-length *EGFR or PPARGC1A*. (Right panel) The PCR products by the fusion gene-specific primers or *GAPDH* primers and ladder marker were run out on agarose gels containing ethidium bromide. Lane M, 100-bp marker; lane 5, cultured human dermal microvascular endothelial cells (EC); lane 6, normal human dermal fibroblasts (FB). The arrow indicates the PCR product of the *EGFR-PPARGC1A*-specific primers (287-bp). The *GAPDH* levels (118-bp) were shown as the control. (**C**) A FISH analysis with human *EGFR-* (green) and *PPARGC1A-*specific (red) probes. NHEKs (left) had two green or red signals (indicated by asterisks), while increased green/red signals were detected in A431 (right). The white arrowheads indicate the fusion signals (yellow). (**D**) The result of a chromosome analysis. (Left) The karyotypes of NHEKs, which were diploid: 46, XX. (Right) A431 had a complex abnormal karyotype and was aneuploid. The karyotype was 72-80,XXX,+1,+3,i(3)(q10)×2,add(4)(p11),add(6)(q13), add(7)(p13),add(8)(p11.2),+11,add(11)(q13)×2,-13,i(14)(q10),-16,add(17)(p11.2),-19,+20,+20,-21,-22,add(22)(q13),+mar1,+mar2,+mar3,+mar4,+mar5×2 [cp20]. The arrows indicate the putative break points. The insets depict chromosomes 4 and 7.

To identify more specific gene changes, we then examined the presence of novel fusion transcripts in A431. We focused on four candidates that were only predicated in A431 and not in NHEKs or DJM-1, using both of two different softwares, deFuse and Fusion Hunter (Supplementary Table S3). Among these, 16 spanning reads and 8 spanning mate pairs indicated fusion between exon16 of *epidermal growth factor receptor (EGFR)* and exon2 of *PPARγ coactivator 1-α (PPARGC1A)*, which are located at 7p12 and 4p15.1, respectively (Fig. 1A and Supplementary Fig. S2A-B). It is noteworthy that the gene fusion caused a frameshift change of *PPARGC1A*, and that the stop codon appears at only 23 amino acids from the breakpoint. Specific primers designed for *EGFR* exon16 and *PPARGC1A* exon2 were used for RT-PCR to validate the finding (Fig. 1B). The expected fragment was amplified by PCR using RNA from A431, but not RNA from NHEKs, DJM-1, cultured human endothelial cells, or dermal fibroblasts. Sanger sequencing of the amplified fragments confirmed the presence of gene fusion between *EGFR* and *PPARGC1A* (Fig. 1A). The above-mentioned gene expression analysis indicated that *EGFR* level in A431 was not substantially up-regulated in comparison to NHEKs (2.95-fold change, q-value = 0.99).

*EGFR-PPARGC1A* was not found in eight normal skin samples or in other tumor tissue specimens, including seven melanoma and five basal cell carcinoma (BCC) by RT-PCR (Supplementary Table S3). Thus, the fusion may be specific to cSCC. Furthermore, the other three candidate fusion genes predicted in A431 by transcriptome analysis (Supplementary Table S3) or *PPARGC1A-EGFR* fusion were not detected in the cell line by RT-PCR validation.

To characterize the rearrangements at the chromosomal level, two-color fluorescence *in situ* hybridization (FISH) analysis was performed (Fig. 1C). Two separate green (*EGFR*) and red (*PPARGC1A*) signals could be detected in NHEKs, while increased single signals were detected in A431, indicating their aneuploidy. The presence of *EGFR*-*PPARGC1A* fusion was confirmed by several yellow signals that were found in all of 100 interphase or metaphase cells determined.

G-banding demonstrated that there was aneuploidy in many chromosomes, including chromosome4 and 7, of A431 (Fig. 1D). However, for example, gene expression analysis showed that only 3 of 1,436 chromosome7 genes were substantially up-regulated, while five genes were down-regulated in A431 cell. Thus, there seems to be no correlation between aneuploid chromosome7 and the expression levels of chromosome7 genes (e.g. due to DNA methylation).

### The clinical significance of *EGFR-PPARGC1A*

**Figure 2:**
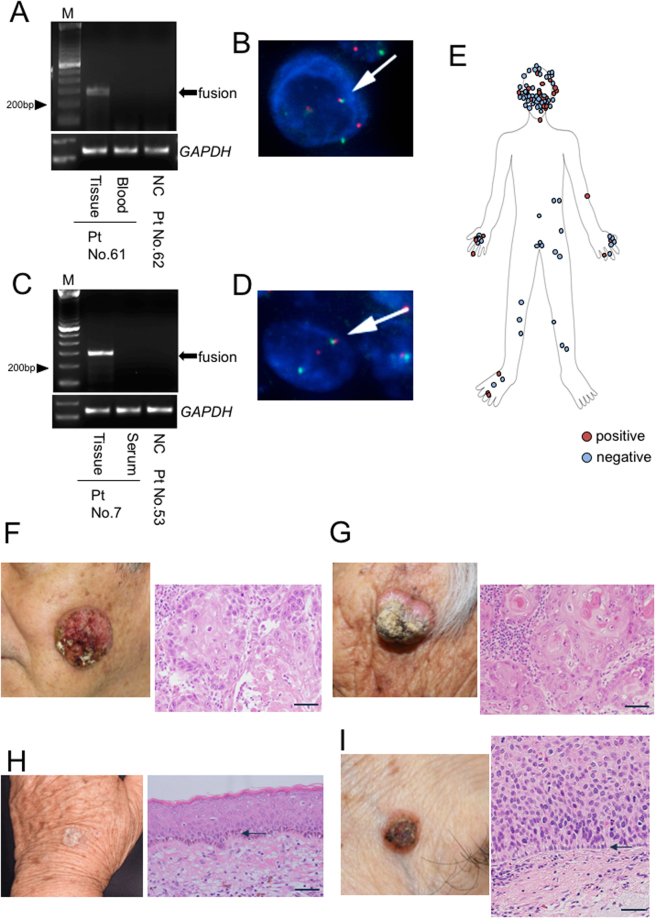
The clinical and histopathological features of cSCC patients with or without *EGFR-PPARGC1A*. (**A**) The RT-PCR-based detection of *EGFR-PPARGC1A*-specific 287-bp fragment in the RNA of cSCC tumor tissue, but not in the blood RNA from the same individual (patient No. 61). M, 100-bp ladder; NC, negative control (patient No.62). The arrow indicates the PCR product of the *EGFR-PPARGC1A*-specific primers. The *GAPDH* levels were shown as the control. (**B**) A FISH analysis of the cSCC tissue sample of patient No.61 with human *EGFR-* (green) and *PPARGC1A-*specific (red) probes. The white arrowhead indicates the fusion signal. (**C**) The RT-PCR-based detection of *EGFR-PPARGC1A*-specific 287-bp fragment in the RNA of cSCC tumor tissue, but not in the serum RNA from the same individual (patient No. 7). M, 100-bp ladder; NC, negative control (patient No.53). The arrow indicates the PCR product of the *EGFR-PPARGC1A*-specific primers. The *GAPDH* levels were shown as the control. (**D**) A FISH analysis of the cSCC tissue sample of patient No.7 with human *EGFR-* (green) and *PPARGC1A-*specific (red) probes. The white arrowhead indicates the fusion signal. (**E**) The tumor distribution of 102 cSCC in our study. The red dots indicate the tumors that were positive for *EGFR-PPARGC1A*; the blue dots indicate those that were negative for the fusion. (**F,G**) Representative clinical pictures and histopathological findings of a patient with (F, patient No.84) and without *EGFR-PPARGC1A* (G, patient No.30). (Left panel) The clinical presentation of the tumors. (Right panel) The results of hematoxylin and eosin staining showing masses of large pleomorphic and hyperchromatic tumor cells with variable keratinization. Nuclear atypia and mitotic figures were also seen. Bar = 50μm. H, (**I**) Representative clinical pictures and histopathological findings of a patient with *EGFR-PPARGC1A*–positive actinic keratosis (H, No.1) and a patient with *EGFR-PPARGC1A*–positive Bowen’s disease (I, No.2). (Left panel) The clinical presentation of the tumors. (Right panel) The results of hematoxylin and eosin staining showing pleomorphic and hyperchromatic tumor cells in the basal layer (F, actinic keratosis) or in stratum spinosum of the epidermis (G, Bowen’s disease). The arrow indicates the basal layer. Bar = 50μm.

To determine whether the *EGFR-PPARGC1A* fusion was recurrent, we evaluated the frequency using RNAs obtained from an increased number of paraffin sections of human tumors using fusion-specific RT-PCR. We screened 102 cSCC specimens and 106 other tumors (BCC, n = 38; melanoma, n = 20; Paget disease, n = 20; dermatofibrosarcoma protuberans, n = 14; and angiosarcoma, n = 14), and detected the fusion gene specifically in 31 cSCC without junctional variability (Table 1). Each of these detections was confirmed by Sanger sequencing and FISH: the sensitivity and specificity of the fusion for cSCC was 30.4% and 100%, respectively (Supplementary Table S4). For example, the amplification by fusion gene-specific primer pair was observed in a cSCC tumor RNA sample from patient No. 61 (Fig. 2A), but not in the peripheral blood cell RNA of the same individual. Thus, the rearrangement was somatically acquired. FISH analysis indicated the single allele localization of chromosomes 4 and 7 as well as their aneuploidy in the tissue DNA specimen of the patient (Fig. 2B). On the other hand, the fusion gene was present in the tumor tissue of patient No.7, but not in the patient’s serum (Fig. 2C), suggesting that the detection of *EGFR-PPARGC1A* was not useful as a serum tumor marker. The gene fusion in the tissue sample was also confirmed by FISH analysis (Fig. 2D). As the clinical significance of the fusion gene, we found that the lesions positive for *EGFR-PPARGC1A* were located in sun-exposed areas substantially more frequently than those negative for the fusion (42.9% vs 0%, P < 0.05, Fig. 2E). Furthermore, three cSCCs of the genital region were negative for the fusion. The other features of the patients with (Fig. 2F) and without the fusion gene (Fig. 2G) were basically similar: both patient groups clinically presented with nodules, tumors, or ulcers. A known genesis, such as burns or traumas, was not correlated with fusion positivity (Table 1). Histopathologically, both poorly- and well-differentiated lesions consisted of pleomorphic and hyperchromatic cells were seen in both of patients with and without fusion gene (Fig. 2F,G). Nuclear atypia and mitotic figures were also found. Both groups included two *in situ* forms of cSCC, actinic keratosis and Bowen’s disease (Fig. 2H,I).

**Table 1:** The clinical and pathological data for the cSCC patients with and without the *EGFR-PPARGC1A* fusion gene.

No.	Age	Sex	Diagnosis	Duration	Region	Size	Appearance	SCC antigen	Genesis	T	N	M	Stage	*EGFR-PPARGC1A*	*FGFR3-TACC3*	*AML4-ALK*	*NSD3-NUT*	*GOLM1-MAK10*
1	79	M	AK	36	cheek	1 cm	erythema			is	0	0	0	+	−	−	−	−
2	88	M	Bowen	0	cheek	1 cm	tumor	1.6		is	0	0	0	+	−	−	−	−
3	93	F	AK	12	cheek	1 cm	tumor	0.9		is	0	0	0	−	−	−	−	−
4	73	M	AK		foot	2 cm	tumor	0.6		is	0	0	0	−	−	−	−	−
5	68	F	AK	48	hand	3.3 × 2.2 cm	tumor	0.5		is	0	0	0	+	−	−	−	−
6	81	M	Bowen		cheek	1.2 × 1.0 cm	erythema			is	0	0	0	−	−	−	−	−
7	90	F	AK	1	head	1.2 cm	tumor	1.1		is	0	0	0	+	−	−	−	−
8	68	M	SCC	24	hand	0.5 cm	ulcer	1.2	burn	1	0	0	I	+	−	−	−	−
9	87	F	SCC		finger	1 cm	tumor	1		1	0	0	I	−	−	−	−	−
10	91	F	SCC		cheek	1 cm	tumor			1	0	0	I	+	−	−	−	−
11	95	F	SCC		cheek	2 cm	tumor	0.9		1	0	0	I	−	−	−	−	−
12	94	F	SCC	60	cheek		tumor			1	0	0	I	−	−	−	−	−
13	86	F	SCC	12	cheek	1.2 × 1.5 cm	tumor			1	0	0	I	−	−	−	−	−
14	82	F	SCC	4	lip	1 cm	tumor	1.1		1	0	0	I	−	−	−	−	−
15	64	M	SCC		cheek	1.2 × 0.8 cm	tumor	0.6	burn	1	0	0	I	−	−	−	−	−
16	54	F	SCC	1	eye lid	2 cm	tumor	0.9		1	0	0	I	−	−	−	−	−
17	82	M	SCC	5	foot	1.1 × 1.0 cm	tumor	1.3		1	0	0	I	−	−	−	−	−
18	81	M	SCC	7	finger	2 × 1.5 cm	tumor	4.2	trauma	1	0	0	I	+	−	−	−	−
19	67	M	SCC	36	inguinal	2 cm	tumor	1		1	0	0	I	−	−	−	−	−
20	77	M	SCC	3	hand	1.0 × 1.2 cm	tumor	1.1		1	0	0	I	−	−	−	−	−
21	86	M	SCC	4	nose	0.5 cm	tumor	2.4		1	0	0	I	−	−	−	−	−
22	89	M	SCC	24	lip	1 cm	tumor	2.2		1	0	0	I	+	−	−	−	−
23	77	M	SCC		hand	2 cm	tumor	1.4		1	0	0	I	−	−	−	−	−
24	66	M	SCC	12	ear	0.7 cm	erosion	1.1		1	0	0	I	+	−	−	−	−
25	72	M	SCC	6	ear	1 cm	tumor	1		1	0	0	I	−	−	−	−	−
26	63	M	SCC	1	nose	0.5 cm	tumor	1.7		1	0	0	I	+	−	−	−	−
27	72	M	SCC	0	cheek	0.8 cm	tumor	1.4		1	0	0	I	−	−	−	−	−
28	73	F	SCC	3	head	2 cm	tumor	0.9		1	0	0	I	−	−	−	−	−
29	91	F	SCC	36	cheek	1 cm	tumor	0.8		1	0	0	I	+	−	−	−	−
30	81	F	SCC	3	cheek	0.3 cm	tumor			1	0	0	I	−	−	−	−	−
31	83	M	SCC	12	cheek	1.1×0.8 cm	tumor	1.7		1	0	0	I	+	−	−	−	−
32	74	M	SCC	3	hand	1 cm	tumor			1	0	0	I	−	−	−	−	−
33	87	F	SCC	6	cheek	1.2×0.8 cm	tumor	1.2		1	0	0	I	−	−	−	−	−
34	82	M	SCC	6	head	1 cm	tumor	0.9		1	0	0	I	+	+	−	−	−
35	72	M	SCC	12	foot	0.5 cm	ulcer	0.8		1	0	0	I	+	−	−	−	−
36	76	M	SCC	6	hand	0.7 cm	tumor	2.9		1	0	0	I	−	−	−	−	−
37	85	F	SCC	600	cheek	2 cm	tumor	1.5		1	0	0	I	+	−	−	−	−
38	71	M	SCC	96	hand	0.8 cm	tumor	1.5		1	0	0	I	−	−	−	−	−
39	94	F	SCC	3	cheek	2 cm	tumor	1.2		1	0	0	I	−	−	−	−	−
40	86	M	SCC	2	hand	1 cm	tumor	2.5		1	0	0	I	−	−	−	−	−
41	80	F	SCC		hand	1 cm	tumor	2.5		1	0	0	I	+	−	−	−	−
42	90	M	SCC	3	cheek	1 cm	tumor	2.2		1	0	0	I	−	−	−	−	−
43	77	F	SCC	0	cheek	0.3 cm	tumor	0.5		1	0	0	I	−	+	−	−	−
44	95	F	SCC	3	ear	1 cm	tumor	3.1		1	0	0	I	+	−	−	−	−
45	76	M	SCC	4	cheek	1.3 × 1.0 cm	tumor	1.2		1	0	0	I	−	−	−	−	−
46	91	F	SCC		hand	1.5 cm	tumor	1.9		1	0	0	I	−	−	−	−	−
47	84	M	SCC	12	ear	0.8 × 0.7 cm	tumor	1		1	0	0	I	−	−	−	−	−
48	72	M	SCC	6	cheek	1.2 × 1.3 cm	tumor	1.1		1	0	0	I	−	−	−	−	−
49	78	M	SCC	2	cheek	1.1 cm	tumor	1.5		1	0	0	I	−	−	−	−	−
50	90	M	SCC	12	head	2 cm	erosion	1.7		2	0	0	II	−	−	−	−	−
51	85	M	SCC		head	4.5 × 4.6 cm	tumor	0.7		2	0	0	II	+	−	−	−	−
52	91	F	SCC	2	inguinal	3.5 × 4 cm	tumor	5.8		2	0	0	II	−	−	−	−	−
53	66	M	SCC	0	foot	5.5 × 2 cm	ulcer	1.5	burn	2	0	0	II	−	−	−	−	−
54	59	M	SCC		foot	2 cm	tumor	0.9		2	0	0	II	+	−	−	−	−
55	76	F	SCC	48	lip	3 cm	tumor	3.2		2	0	0	II	−	−	−	−	−
56	76	M	SCC	48	head	2.1 × 1.3 cm	tumor	0.6	burn	2	0	0	II	+	−	−	−	−
57	78	M	SCC	4	elbow	10 cm	tumor	1.4	trauma	2	0	0	II	+	−	−	−	−
58	85	M	SCC	1	finger	2.5 × 2.0 cm	erosion	4.3		2	0	0	II	+	−	−	−	−
59	91	F	SCC		nose	2.1 × 1.6 cm	tumor	1.4		2	0	0	II	−	−	−	−	−
60	81	M	SCC	3	ear	2 × 2 cm	tumor	2.3		2	0	0	II	−	−	−	−	−
61	75	F	SCC	3	lip	2 cm	tumor	0.8		2	0	0	II	+	−	−	−	−
62	72	M	SCC	1	foot	4 cm	ulcer	3	trauma	2	0	0	II	−	−	−	−	−
63	53	M	SCC	6	lip	2 cm	tumor	0.8		2	0	0	II	−	−	−	−	−
64	76	M	SCC		hand	2 cm	tumor	1.2		2	0	0	II	−	−	−	−	−
65	71	F	SCC	120	inguinal	2 cm	ulcer	1.7		2	0	0	II	−	−	−	−	−
66	85	M	SCC	1	neck	2 cm	tumor			2	0	0	II	+	−	−	−	−
67	90	M	SCC	120	abdomen	3.5 × 2.5 cm	tumor	0.8		2	0	0	II	−	−	−	−	−
68	59	F	SCC	48	abdomen	8 cm	tumor	11.5		2	0	0	II	−	−	−	−	−
69	69	M	SCC	24	knee	8 cm	scar	3.1	burn	2	0	0	II	−	−	−	−	−
70	61	M	SCC	4	joe	2 cm	tumor	1.5		2	0	0	II	−	−	−	−	−
71	79	M	SCC	48	head	2 cm	tumor	2.4	burn	2	0	0	II	−	−	−	−	−
72	87	M	SCC	24	hand	2 cm	tumor	1.3		2	0	0	II	−	−	−	−	−
73	84	F	SCC	6	foot	2 cm	tumor	0.5		2	0	0	II	−	−	−	−	−
74	89	F	SCC	24	finger	2 × 1.5 cm	tumor	0.8		2	0	0	II	−	−	−	−	−
75	91	F	SCC	2	foot	2 × 1.5 cm	tumor	1.3		2	0	0	II	+	−	−	−	−
76	70	M	SCC	60	head	3 cm	tumor	1.4	burn	2	0	0	II	−	−	−	−	−
77	85	F	SCC	4	head	2.5 cm	tumor	1.2		2	0	0	II	+	−	−	−	−
78	94	M	SCC	12	foot	7.5 cm	erosion	1.9		2	0	0	II	−	−	−	−	−
79	56	M	SCC	0.5	nose	2.5 cm	tumor	1.4		2	0	0	II	−	−	−	−	−
80	83	M	SCC	12	ear	2.9 × 2.0 cm	tumor	0.7		2	0	0	II	−	−	−	−	−
81	88	F	SCC	24	head	2.5 × 1.7 cm	tumor	1.1	burn	2	0	0	II	−	−	−	−	−
82	78	F	SCC		cheek	2.5 cm	tumor	0.8		2	0	0	II	−	−	−	−	−
83	69	M	SCC	60	back	2 cm	tumor	9.4		2	1	0	III	−	−	−	−	−
84	75	M	SCC	3	cheek	4 cm	tumor	2		2	1	0	III	+	−	−	−	−
85	91	F	SCC	12	head	2.7 cm	tumor	1.8		2	1	0	III	−	−	−	−	−
86	94	F	SCC	6	head	4 × 3 cm	tumor	2.9		3	0	0	III	−	−	−	−	−
87	76	M	SCC	3	lip	4.5 × 2 cm	tumor	1.4		2	1	0	III	+	−	−	−	−
88	44	M	SCC	3	foot		tumor	2	burn	4	1	0	III	−	−	−	−	−
89	73	F	SCC	3	head	0.8 × 0.8 cm	ulcer	1.8		1	1	0	III	−	−	−	−	−
90	83	M	SCC	1	cheek	2.2 cm	tumor	2.4		x	1	0	III	+	−	−	−	−
91	82	F	SCC	1	cheek	3 × 2 cm	tumor	1.5		3	0	0	III	+	−	−	−	−
92	63	F	SCC	0	head			0.8		3	0	0	III	−	−	−	−	−
93	71	F	SCC	0	cheek			1.1		x	1	0	III	−	−	−	−	−
94	84	M	SCC	36	ear		ulcer	1.4		4	0	0	III	−	−	−	−	−
95	54	F	SCC	3	head	8 × 7 cm	tumor	3.9	burn	4	0	0	IV	−	−	−	−	−
96	68	M	SCC	6	foot	21.1 × 7.8 cm	tumor	1.3		x	2	0	IV	−	−	−	−	−
97	83	M	SCC	1	foot	0.5 × 0.3 cm	tumor	0.5		1	1	1	IV	+	−	−	−	−
98	80	M	SCC	0	cheek			3.7		1	2	0	IV	−	−	−	−	−
99	81	M	SCC	48	foot		tumor			x	1	1	IV	−	−	−	−	−
100	95	F	SCC	8	cheek	2 cm	ulcer			3	x	x	X	−	−	−	−	−
101	71	M	SCC		nose		ulcer						X	−	−	−	−	−
102	94	F	SCC	12	cheek	2 cm	tumor	1.2		2			X	−	−	−	−	−

AK, actinic keratosis; Bowen, Bowen’s disease; SCC, squamous cell carcinoma.

Several studies have reported the detection of fusion genes in SCC of other organs (*AML4-ALK* in lung SCC, *FGFR3-TACC3* in head and neck SCC, *NSD3-NUT* in NUT midline carcinoma, and *GOLM1-MAK10* in esophageal SCC). We determined their frequency in our cSCC patients. Two cases were positive for *FGFR3-TACC3* fusion gene (the patients were also positive for *EGFR-PPARGC1A* or *ADCK4-NUMBL*), but no other fusion genes were detected (Table 1).

### The functional analysis of *EGFR-PPARGC1A*

**Figure 3:**
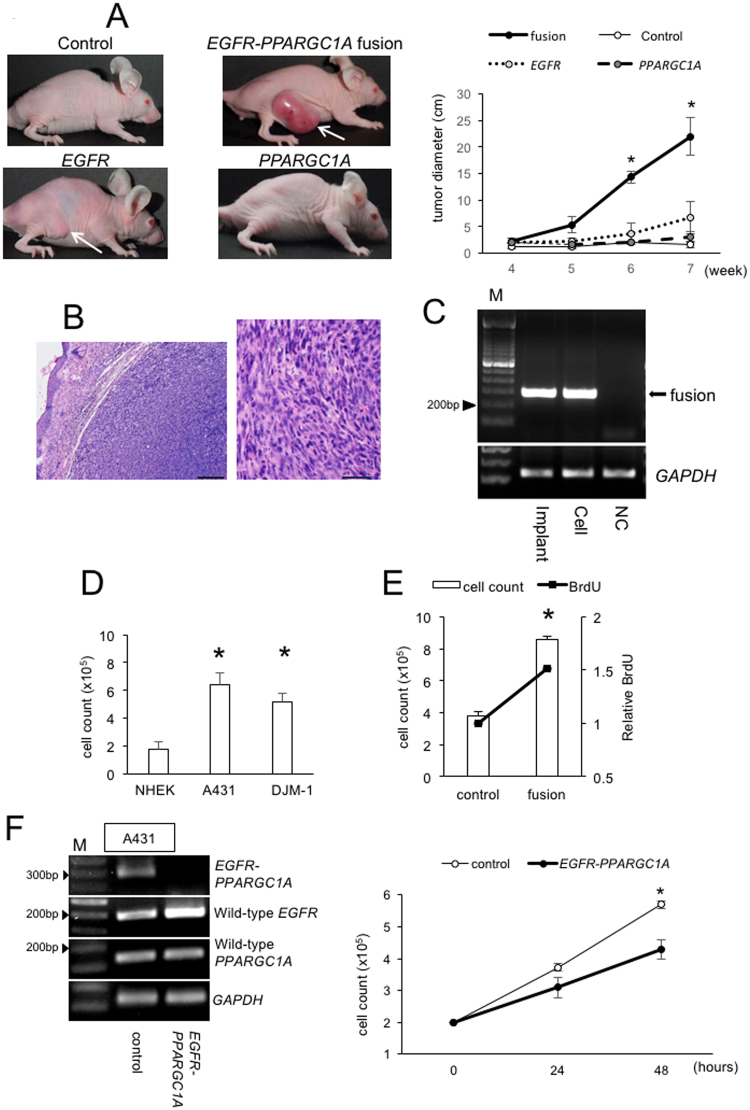
The forced overexpression of EGFR-PPARGC1A in NIH3T3. (**A**) NIH3T3 cells transfected with the control vector, *EGFR-PPARGC1A* fusion gene, full-length *EGFR* gene, or full-length *PPARGC1A* gene were injected into mouse skin (1 × 10^5^ cells). (Left) After five weeks, tumor formation (white arrow) was observed in 11 of 14 mice that had been injected with cells overexpressing the fusion gene and 5 of 7 mice injected with cells overexpressing full-length *EGFR*. Tumor formation was not detected in the 7 mice injected with *PPARCG1A*-overexpressing cells or 12 mice with control vector. (Right) Tumor diameter was determined at the indicated times. Data are mean values and SD. *P < 0.05 (n = 3). (**B**) Hematoxylin and eosin staining of the implants of NIH3T3 cells that had been transfected with *EGFR-PPARGC1A*. The tumor cells were pleomorphic and hyperchromatic. Mitotic figures were also seen. (Left) Low magnification, bar = 200 μm, (Right) High magnification, bar = 50 μm. (**C**) The RT-PCR-based detection of *EGFR-PPARGC1A*-specific 287-bp fragments in RNA from implanted NIH3T3 cells that had been transfected with the fusion gene (Implant) and cultured cells from the implants (Cell). The arrow indicates the PCR products of the fusion gene-specific primers. M, 100-bp ladder; NC, PCR-negative control using NIH3T3 transfected with the control vector. (**D**) NHEKs, A431, and DJM-1 (1 × 10^5^ cells) were plated and grown in six-well plates. After 72 hours, the cells were detached from the wells by trypsin treatment and counted. *P < 0.05 in comparison to NHEKs (n = 3). (**E**) For the proliferation analysis, NIH3T3 cells that were stably transfected with a lentiviral control or *EGFR-PPARGC1A* fusion gene were counted as described in the Fig. 4D. The cells were also labeled with BrdU and analyzed by ELISA. The white bars indicate the cell numbers, and the black line represents the relative absorbance as determined by BrdU ELISA. *P < 0.05 (n = 3). (**F**) The expression of *EGFR-PPARGC1A* was knocked down in A431 using CRISPR/Cas9 system targeting their junctions. (Left) To show the transfection efficiency, the PCR products obtained using the primer pairs for *EGFR-PPARGC1A*, wild-type *EGFR, or* wild-type *PPARGC1A* were run out on agarose gels containing ethidium bromide. The *GAPDH* levels were shown as the control. M, 100-bp ladder. (Right) Cell number was determined at the indicated times. Data are mean values and SD. *P < 0.05 (n = 3).

To clarify the function of EGFR-PPARGC1A protein (Supplementary Fig. S3A), the fusion gene was amplified by using PCR and cloned into lentiviral vector. We first tried to overexpress the fusion protein in NHEKs by lentiviral transfection; however, the cells were very fragile and stopped proliferating after transfection. Instead, we performed a tumorigenicity assay using NIH3T3, according to the methods of a previous study^12^. NIH3T3 that were stably transfected with the control vector, *EGFR-PPARGC1A*, full-length wild-type *EGFR*, or full-length *PPARGC1A* showed no apparent differences in cell shape (Supplemental Fig. S3B). Following subcutaneous implantation, tumorigenesis was observed in 11 of 14 samples of NIH3T3 transfected with the fusion gene, while the 12 samples transfected with the control vector did not cause tumorigenesis (Fig. 3A). The histopathological findings of the tumor tissue showed a mass of atypical, pleomorphic and hyperchromatic cells with mitosis (Fig. 3B), which were similar to the histopathological features of cSCC (Fig. 2F). We confirmed that the tumor tissues, as well as the cells cultured from them, were positive for *EGFR-PPARGC1A* by RT-PCR with fusion-specific primers (Fig. 3C). Tumor formation was also observed in 5 of the 7 mice with cells that overexpressed full-length *EGFR* gene, although their tumor size was smaller than fusion gene-overexpressing cells (Fig. 3A). Any of the 7 mice with cells overexpressing *PPARCG1A* did not show tumorigenesis. Taken together, these results suggest that *EGFR-PPARGC1A* drives tumorigenesis, probably through EGFR signaling.

We attempted to address the mechanism by which *EGFR-PPARGC1A* causes tumorigenesis. The cell numbers of A431 and DJM-1 were substantially increased in comparison to NHEKs (Fig. 3D). Considering the reported potential role of mitogenesis in the development of cSCC^13^, we focused on the possibility that the proliferation of A431 was activated by *EGFR-PPARGC1A*. The stable overexpression of the fusion gene in NIH3T3 led to a substantial increase in the cell numbers and the incorporation of BrdU (Fig. 3E). On the other hand, *EGFR* siRNA inhibited the expression of the fusion gene (Supplementary Fig. S4A), as well as the full-length wild-type *EGFR* in A431, and substantially reduced the cell number in comparison to control siRNA (Supplementary Fig. S4A), indicating therapeutic value of the siRNA. For the more specific inhibition of fusion gene, *EGFR-PPARGC1A* was knocked down using CRISPR/Cas9 system targeting their junctions (Fig. 3F). The expression levels of full-length *EGFR* or *PPARGC1A* were not affected. The inhibition of *EGFR-PPARGC1A* substantially reduced cell number of A431, demonstrating that *EGFR-PPARGC1A* is required for the growth of A431.

**Figure 4:**
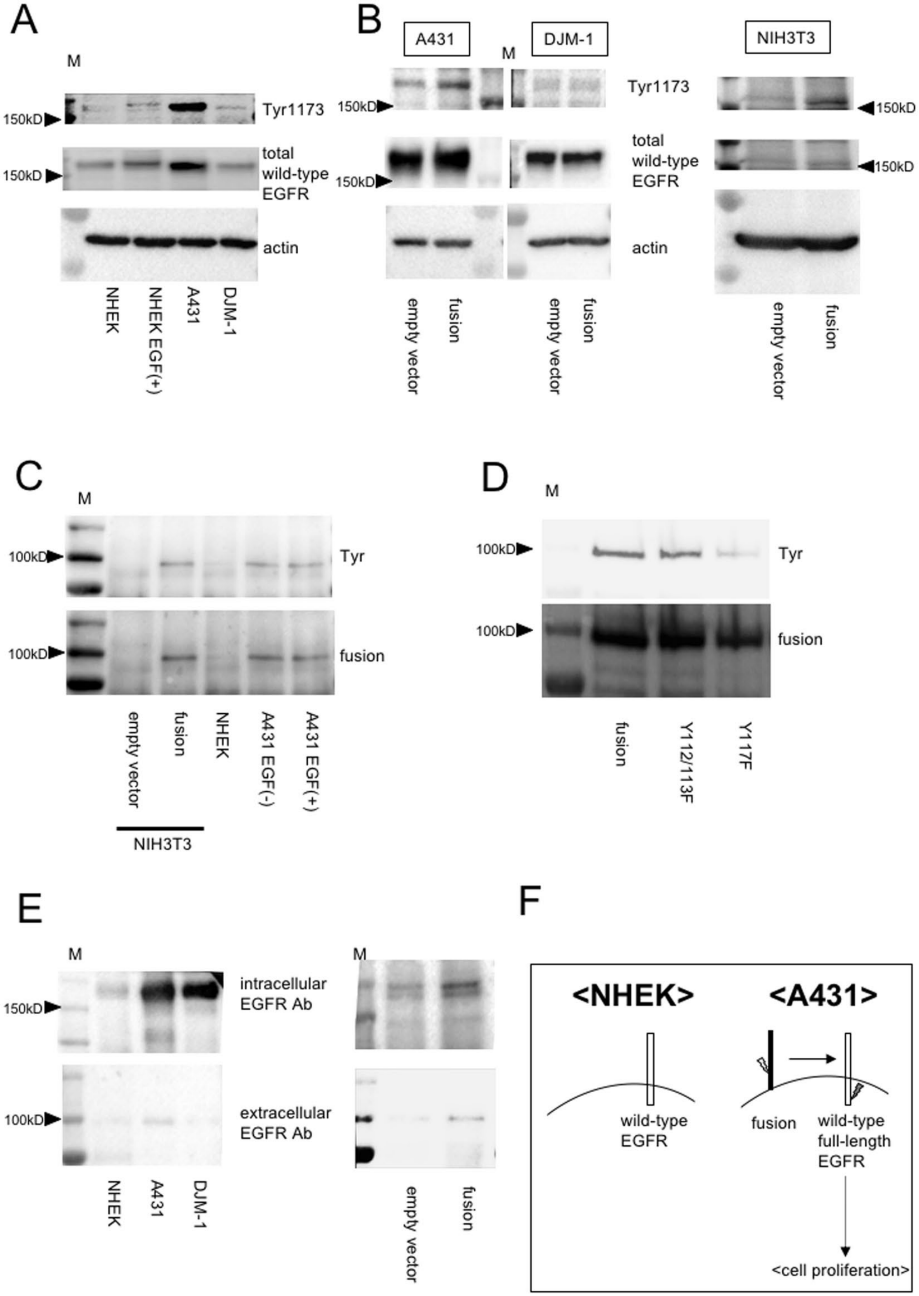
The role of the *EGFR-PPARGC1A* fusion gene in the constitutive phosphorylation of wild-type EGFR in A431. (**A**) Immunoblots of cell lysates with antibodies to phospho-EGFR Tyr1173 and total wild-type full-length EGFR. NHEKs were treated with or without EGF (100ng/μl) for 15 minutes before lysis. Actin was shown as the loading control. M, molecular marker. Cropped images were displayed and original blots are shown in the Supplementary Fig. S5. (**B**) Lysates were obtained from A431, DJM-1 (Left) or NIH3T3 (Right) stably transfected with empty vector or the *EGFR-PPARGC1A* fusion gene. Immunoblotting was performed using antibodies against phospho-EGFR Tyr1173, total wild-type EGFR, and actin. M, molecular marker. Cropped images were displayed and original blots are shown in the Supplementary Fig. S6. (**C**) Cell lysates were immunoprecipitated with antibody to the extracellular domain of EGFR, followed by immunoblotting with anti-phosphotyrosine (4G10) antibody (Tyr). The same membrane was then stripped and reprobed with anti-EGFR extracellular domain antibody to determine the abundance of total EGFR-PPARGC1A fusion protein. A431 were treated with or without EGF for 3 hours before lysis. M, molecular marker. Cropped images were displayed and original blots are shown in the Supplementary Fig. S7. (**D**) The *EGFR-PPARGC1A* fusion gene or the fusion genes with the indicated mutations were transfected into NIH3T3 cells using lentiviral constructs. Cell lysates were immunoprecipitated with antibody to the extracellular domain of EGFR, followed by immunoblotting with anti-phosphotyrosine (4G10) antibody (Tyr). The same membrane was stripped and reprobed with anti-EGFR extracellular domain antibody. Cropped images were displayed and original blots are shown in the Supplementary Fig. S8. (**E**) Immunoblots of lysates from NHEKs, A431, or DJM-1 (left) and NIH3T3 stably transfected with empty vector or the *EGFR-PPARGC1A* fusion gene (right), following immunoprecipitation with antibody (Ab) to the intracellular domain of wild-type EGFR. The blots were incubated with antibody to the extracellular domain of EGFR to detect the EGFR-PPARGC1A fusion protein. M, molecular maker. Cropped images were displayed and original blots are shown in the Supplementary Fig. S9. (**F**) Our hypothetical model of the role of the *EGFR-PPARCG1A* fusion gene in A431. The EGFR-PPARGC1A protein shows constitutive tyrosine phosphorylation, but cannot transduce cellular signals due to the lack of an intracellular domain. Instead, the fusion protein phosphorylates the wild-type full-length EGFR through their interaction, which stimulates cellular proliferation without ectopic stimulation.

Next, to investigate the mechanism underlying the activation of A431 proliferation by *EGFR-PPARGC1A*, we focused on EGFR signaling, because tyrosine phosphorylation of the wild-type full-length EGFR has been well-implicated in the keratinocyte proliferation^14^. In many of the tyrosine phosphorylation residues of wild-type EGFR, Y1173 (the major phosphorylation site) was hardly phosphorylated in NHEKs, but its phosphorylation was induced by ectopic EGF stimulation (Fig. 4A). On the other hand, as described previously^15^, unstimulated A431 or DJM-1 showed strong or weaker constitutive Y1173 phosphorylation, respectively, which is consistent with their cellular proliferation activity (Fig. 3D). The Y1173 phosphorylation levels in A431 were further enhanced by the viral overexpression of *EGFR-PPARGC1A* fusion gene (Fig. 4B), but not in DJM-1. Furthermore, NIH3T3 is reported to express a small amount of wild-type EGFR^16^, and its phosphorylation level was also induced by the fusion gene (Fig. 4B). Accordingly, it is possible that the constitutive tyrosine phosphorylation of wild-type full-length EGFR in A431 may be induced by the presence of fusion gene.

*NUP214-ABL1* fusion causes its own phosphorylation, which contributes to the pathogenesis of T-cell acute lymphoblastic leukemia^17^. Thus, we determined the phosphorylation levels of EGFR-PPARGC1A protein (Supplementary Fig. S3A). Immunoprecipitation with the antibody for the extracellular domain of wild-type EGFR revealed that the fusion protein (approximately 100kDa) could only be detected in NIH3T3 overexpressing the fusion gene and A431, and showed tyrosine phosphorylation (Fig. 4C). Ectopic EGF stimulation did not affect the phosphorylation of the fusion. The antibody for EGFR extracellular domain did not react with wild-type full-length EGFR, while the detection of the fusion protein was confirmed by immunoprecipitation with antibody against His-tag attached to the fusion protein (Supplementary Fig. S4B). A Y117F substitution mildly reduced the tyrosine phosphorylation levels of the fusion protein (Fig. 4D), but Y112/113 substitution did not. Thus, the phosphorylation at least partly involves Y117. On the other hand, although EGFR-PPARGC1A also has several serine/threonine residues, serine/threonine phosphorylation was not detected.

Given that wild-type EGFR phosphorylation is correlated with their dimerization^14^, we next hypothesized that the EGFR-PPARGC1A fusion protein (approximately 100kDa) can form a dimer with wild-type full-length EGFR (approximately 170kDa), and that they would interact with each other. Immune complexes generated with antibody to EGFR intracellular domain from unstimulated A431 lysates contained fusion protein (Fig. 4E), which was detected using EGFR extracellular domain antibody: because the fusion protein lacks an intracellular domain according to NCBI database (Supplementary Fig. S3A), this result suggested an interaction between wild-type EGFR and the fusion protein. Similarly, the interaction was found in NIH3T3 by overexpressing fusion gene (Fig. 4E).

Taken together, our hypothetical model regarding the role of the fusion gene in A431 is shown in Fig. 4F. The EGFR-PPARGC1A protein shows constitutive tyrosine phosphorylation, but cannot transduce signals inside the cells due to the lack of an intracellular domain. Instead, the fusion protein phosphorylates wild-type full-length EGFR by dimerization, which stimulates cellular proliferation via the activation of wild-type EGFR.

On the other hand, Lin *et al*. noted that A431 is sensitive to gefitinib, an EGFR tyrosine kinase inhibitor, but that other SCC cell lines are not^18^. Consistently, we also found that the cell number of A431 was reduced by gefitinib, but that DJM-1 was not sensitive to gefitinib treatment (Supplementary Fig. S4C). Gefitinib inhibited the phosphorylation levels of wild-type full-length EGFR as described previously^19^, but not the fusion protein phosphorylation (Supplementary Fig. S4D). Accordingly, the effect of gefitinib seems to occur via wild-type EGFR.

## Discussion

Recently, we first reported the presence of novel *NUP160-SLC43A3* fusion gene in angiosarcoma^9^. In addition, we previously found a case of dermatofibrosarcoma protuberans that was positive for novel *COL1A1* exon 14/*PDGFB* fusion^20^.

In this study, we performed global gene expression analysis and global mutation detection using A431 and DJM-1. Because these are the most commonly-used SCC cell lines, these analyses will be useful for future studies. Transcriptome sequencing is powerful tool for fusion gene detection as well as gene expression analysis and discovery of point mutations. Fusion genes are often found in mesenchymal tumors or sarcomas, and in-frame fusion genes are the most common. However, *EGFR-PPARGC1A* fusion gene in A431 is characterized by its presence in epithelial carcinoma and the frameshift of its 3′ fusion partner. A similar well-characterized fusion gene in epithelial tumor, *MYB-NFIB* fusion, was reported in adenoid cystic carcinoma^21^. Furthermore, several studies have reported out-of-frame fusion genes in malignancies, including acute myeloid leukemia^22^.

Wild-type full-length EGFR is a transmembrane glycoprotein that acts as a receptor for EGF. Through ligand binding, the EGFR dimerized and phosphorylated, which led to cellular proliferation. EGFR signaling is thought to play a central role in the pathogenesis of SCC in various organs including cSCC, and its constitutive phosphorylation has been reported in A431^23^. It is noteworthy that a mutated EGFR lacking an extracellular domain, so-called ΔEGFR, was found to contribute to the pathogenesis of glioblastoma^24^. When it is considered that *EGFR-PPARGC1A* causes frameshift, although PPARGC1A acts as a transcriptional coactivator that regulates genes involved in energy metabolism, it is not likely to express the function in the fusion protein. Instead, EGFR-PPARGC1A may function via phosphorylation, probably through the truncation of EGFR intracellular domain and conformation change or through an interaction with wild-type endogenous EGFR, which are novel insights of EGFR function.

*EGFR-PPARGC1A* was found in keratinocytes, but also induced tumor formation in mouse fibroblasts NIH3T3, suggesting its potent tumorigenicity. The increased phosphorylation and dimer formation of wild-type EGFR was also seen in NIH3T3 with the overexpression of the fusion gene, which supports our hypothesis. *EGFR-PPARGC1A*-negative DJM-1 also showed mild wild-type EGFR phosphorylation, but it was not further induced by *EGFR-PPARGC1A* overexpression. Thus, the mild phosphorylation may be mediated by different mechanism in DJM-1.

Other fusion genes that have been reported in SCCs of other organs were rare in cSCC. This may be because cSCC differs from other SCCs in terms of the strong correlation with chronic sun exposure, and *EGFR-PPARGC1A* may be the fusion gene that is associated with chronic sun exposure. This hypothesis is supported by the detection of *EGFR-PPARGC1* in cSCCs of sun-exposed areas. Furthermore, genital SCC, which is often correlated with papilloma virus infection, was negative for the fusion. Increasing attention is being paid to the role of ultraviolet irradiation in skin carcinogenesis, and the fusion gene may be the link between sun-exposure and carcinogenesis.

The diagnosis of atypical, early-stage, or well-differentiated cSCC is sometimes challenging because of the lack of specific cell markers. The detection of *EGFR-PPARGC1A* by RT-PCR or variant assays may be useful for the diagnosis of such challenging cases or the evaluation of surgical margins, because the fusion can be detected at the *in situ* stage (actinic keratosis or Bowen’s disease). Furthermore, *EGFR* siRNA, *EGFR-PPARGC1A* CRISPER/Cas9, or gefitinib substantially decreased the cell number of A431, indicating that full-length wild-type *EGFR* and/or the *EGFR-PPARGC1A* fusion may represent potential therapeutic targets. Lung SCC with or without *EGFR* mutations has been treated using various EGFR-targeted therapies, such as gefitinib, erlotinib, or afatinib, because EGFR signaling is the key pathway as described above. In addition, cetuximab is reported to have therapeutic effects against cSCC, although its mechanisms are still unknown^25^. Our results may become the basis for the use of EGFR-targeted drugs for cSCCs. The efficacy of EGFR-targeted drugs may vary due to the phosphorylation state of wild–type EGFR of each cSCC lesion, and may be predicted by the detection of the fusion gene. Such personalized medicine would be helpful for avoiding side effects in elderly patients.

As the limitation of this study, there is no junction variability in *EGFR-PPARGC1A*. This is different from other well-established fusions such as *BCR-ABL* in chronic myelogenous leukemia, *TMPRSS2-ERG* in prostate cancer, or *EWS-FLI1* in Ewing’s sarcoma with junctional variability. However, FISH, functional experiments, and tumorigenicity assay indicated *EGFR-PPARGC1A* is the causative fusion gene. Further studies will be needed to determine the mechanism through which *EGFR-PPARGC1A* in cSCC regulate tumor formation, which may lead to a better understanding of the pathogenesis of cSCC, new diagnostic methods, and new targeted cancer therapies.

## Materials and Methods

This study was approved by the medical ethics research committee of Kumamoto University and written informed consent was obtained from each patient. This informed consent include a statement about publication of images in an online open-access publication. We confirmed that all methods were carried out in accordance with the relevant guidelines and regulations.

### Cell culture

A431 (an established human cSCC cell line) and NIH3T3 were obtained from American Type Culture Collection (ATCC, Manassas, VA)^26^. DJM-1 (another cSCC cell line) was purchased from RIKEN BRC (Tsukuba, Japan). Normal human epidermal keratinocytes (NHEKs) were from Lonza (Walkersville, MD).

### RNA isolation

The RNA was extracted from paraffin-embedded sections using RNeasy FFPE kit (Qiagen, Valencia, CA). For the transcriptome sequencing, high quality RNAs were obtained from cultured cells using RNeasy mini kit (Qiagen)^27^.

### Library preparation and sequencing

The transcriptome analysis was performed using Illumina TruSeq RNA Sample Preparation Kit (San Diego, CA) according to the protocol provided by Riken Genesis (Kanagawa, Japan)^27^. In brief, poly-A-containing mRNA was purified from high quality total RNA using poly-T oligo-conjugated magnetic beads. The mRNA was fragmented by divalent cations, and the cleaved RNA fragments were transcribed into first-strand cDNA using random primers and SuperScriptII Reverse Transcriptase (Invitrogen, Carlsbad, CA), followed by second strand cDNA synthesis using DNA polymerase I and RNase H. The cDNA fragments were then subjected to an end repair process, A-base addition, and the ligation of adapters, to convert them into a library of template molecules suitable for the subsequent cluster generation. The product was purified and enriched by PCR to create final cDNA library. The library was sequenced in a paired-end 100-bp configuration on Illumina HiSeq2000 platform. Adapter sequences and low-quality sequences were eliminated using Cutadapt (v1.0) software^28^. After performing quality control, the poly-A/T sequences were also eliminated using PRINSEQ (v0.16)^29^.

### Transcriptome analysis

The cleaned and trimmed reads were aligned to the reference human sequence (GRCh37/hg19) using TopHat (v1.4.0), a fast splice junction mapping software that uses Bowtie alignment to align reads^30–34^. The mapped reads were assembled using Cufflinks (v2.0.0) software, and the transcripts across all samples were merged using Cuffmerge program. The reference GTF annotation file used in Cufflinks was downloaded from iGenomes database.

The differential expression between samples was analyzed using Cuffdiff software by calculating the fragments per kilobase per million map reads (FPKM) and by testing the statistical significance of the differences. A bioinformatic analysis was performed to detect mutations using Samtools (v1.0). Potential gene fusion transcripts were identified and filtered using deFuse (v0.61) and Fusion Hunter (v1.4).

### RT-PCR

RT-PCR was performed for 40 cycles (denaturation for 15 seconds at 94 °C, annealing for 30 seconds at 55 °C, and extension for 30 seconds at 68 °C) using total RNAs, primers, and SuperScript One-Step RT-PCR System with Platinum Taq Polymerase (Invitrogen). The sequences of the *EGFR-PPARGC1A*-specific primers were: (forward) ATCCAGTGTGCCCACTACATTG and (reverse) GCTGTCTGTATCCAAGTCGTTC. *GAPDH* primers were (forward) GCACCGTCAAGGCTGAGAAC and (reverse) TGGTGAAGACGCCAGTGGA. The PCR products were run on agarose gels containing ethidium bromide. The detection of other fusion genes was performed as previously described^35–37^.

### Fluorescence *in situ* hybridization (FISH)

A FISH analysis of cultured cells was performed according to the protocol provided by Nihon Gene Research Laboratories (NGRL, Miyagi, Japan)^27^. The cells were fixed with freshly made Carnoy’s solution. The fixed cells were dropped onto glass slides and air-dried. The probes were designed by Empire Genomics (Buffalo, NY): the probe for *EGFR* was labeled with fluorescein, whereas that for *PPARGC1A* was labeled with ROX. The slides were treated with probes, and were denatured by heating, followed by incubation at 37 °C overnight for hybridization.

Dual-color FISH analysis of formalin-fixed, paraffin-embedded tissue sections was performed by Chromosome Science Lab (Sapporo, Japan)^38^. Sections were deparaffinized, washed, digested in pepsin solution and dehydrated. Bacterial artificial chromosome clones RP11-752O3 and RP11-118N21 were labeled with SpectrumRed-dUTP and were used as probes for *PPARGC1A*. RP11-81B20, RP11-815K24 and RP11-1055P9 were labeled with SpectrumGreen-dUTP and used as probes for *EGFR*. The probes were applied to the sections and were simultaneously denatured. Hybridization was carried out at 37 °C overnight.

The nuclei were stained with DAPI, and fluorescence was detected by fluorescence microscopy.

### G-banding

A karyotype analysis using cultured cells was carried out by G-banding according to the protocol of NGRL^27^. In brief, cultured cells on the slides were treated with 0.2% trypsin in Hanks' balanced salt solution. The slides were then washed with phosphate-buffered saline, and stained with Giemsa solution.

### Virus generation and transfection

pCMV-VSV-G-RSV-Rev, CSII-EF-RfA, and pHIVgp, which are essential for lentiviral gene expression, were kindly donated by Dr. Hiroyuki Miyoshi (RIKEN, Wako, Japan)^39,40^. cDNA fragments of full-length *EGFR*, *PPARGC1A*, or *EGFR-PPARGC1A* fusion gene were amplified by PCR and cloned into CSII-EF-RfA. Substitution mutations were generated using Quick Change lightning site-directed mutagenesis kit (Agilent Technologies, Santa Clara, CA) and were confirmed by sequencing. Lentiviral vector-mediated gene transfer was performed as described previously^41^.

### Tumorigenicity assay

NIH3T3 cells were implanted into six-week-old female athymic nu/nu mice (BALB/cAJcl-*nu/nu*, CLEA, Tokyo, Japan) by skin injection using a 25-gauge needle^12^. The implants were removed at eight weeks after xenografting, fixed in 10% buffered formalin, embedded in paraffin, and sliced into sections. Protocols of animal experiments were approved by the Committee on the Animal Research at Kumamoto University.

### Cell count and BrdU ELISA

Cultured cells were detached from the wells by trypsin treatment and counted using TC20 Automated Cell Counter (Bio-Rad Laboratories, Hercules, CA)^42^. The proliferation activity of cells was confirmed using Cell proliferation ELISA BrdU kit (Roche, Basel, Switzerland), as described previously^43,44^.

### siRNA transfection

siRNA against *EGFR* was purchased from Qiagen. For reverse transfection, siRNAs mixed with Lipofectamine RNAiMAX (Invitrogen) was added when the cells were plated: the plates were then incubated at 37°C in 5% CO_2_.

### CRISPR/Cas9

Custom CRISPR RNAs targeting the junctions of *EGFR-PPARGC1A* (CAACGAAUGGUGUGCUGCUCGUUUUAGAGCUAUGCUGUUUUG) and trans-activating RNAs (5′-AACAGCAUAGCAAGUUAAAAUAAGGCUAGUCCGUUAUCAACUUGAAAAAGUGGCACCGAGUCGGUGCUUUUUUU-3’) were co-transfected into cultured cells with DharmaFECT Duo Transfection Reagent (Dharmacon)^45^.

### Western blotting

Protein was extracted from harvested cells with RIPA buffer (Nacalai tesque, Kyoto, Japan). Immunoblotting was performed with antibodies for phosphotyrosine, phosphoserine/threonine (Abcam, Cambridge, United Kingdom), EGFR, phosphoEGFR, or β-actin (Santa Cruz Biotechnology, Santa Cruz, CA)^46^.

### Immunoprecipitation

Cells were lysed in RIPA buffer with Phosphatase Inhibitor Cocktail (Nacalai tesque). The lysates were precleared with protein A/G-agarose (Santa Cruz Biotechnology) and control IgG at 4 °C for 30 minutes.

The lysates were then incubated with anti-EGFR antibodies (Santa Cruz Biotechnology) at 4 °C, followed by incubation with protein A/G-agarose. The immunoprecipitated proteins were washed with RIPA buffer, and were subjected to electrophoresis.

### Statistical analysis

Bar graphs were created using the means+standard deviation (SD) from at least three experiments. Mann-Whitney U-test was used to compare median values, while Fisher’s exact probability test was used to compare frequencies. P values of <0.05 were considered to indicate statistical significance.

The P values in the differential expression analysis of the transcriptome sequencing were calculated using the Cuffdiff software^47^. Q values, estimated false discove ry rates using Benjamini-Hochberg procedure, were also calculated.

## Electronic supplementary material

Supplementary Data
