# Supplementary material for: Chronic sun exposure-related fusion oncogenes EGFR-PPARGC1A in cutaneous squamous cell carcinoma
Source: Sci Rep. 2017 Oct 4;7:12654. doi: 10.1038/s41598-017-12836-z (PMC5627299; doi:10.1038/s41598-017-12836-z)

**Supplementary Data**

**Chronic sun exposure-related fusion oncogenes EGFR-PPARGC1A in cutaneous squamous cell carcinoma**

Sho Egashira, Masatoshi Jinnin*, Manami Ajino, Naoki Shimozono, Sayo Okamoto, Yukino Tasaki, Ayaka Hirano, Maho Ide, Ikko Kajihara, Jun Aoi, Miho Harada, Toshikatsu Igata, Shinichi Masuguchi, Satoshi Fukushima, Hironobu Ihn

**Affiliations:**

Department of Dermatology and Plastic Surgery, Faculty of Life Sciences, Kumamoto University, 1-1-1 Honjo, Chuo-ku, Kumamoto 860-8556, Japan

**Supplementary Table S1 …………………………………………………….Page 2**

**Supplementary Table S2 ……………………………………………………..Page 4**

**Supplementary Table S3 ……………………………………………………..Page 5**

**Supplementary Table S4 ……………………………………………………..Page 6**

**Supplementary Figure S1 ……………………………….…………….………..Page 7**

**Supplementary Figure S2 ….…….…………………….……………………....Page 9**

**Supplementary Figure S3 ….…….…………………….……………………....Page 10**

**Supplementary Figure S4 ….…….…………………….……………………....Page 11**

**Supplementary Figure S5 ….…….…………………….……………………....Page 13**

**Supplementary Figure S6 ….…….…………………….……………………....Page 14**

**Supplementary Figure S7 ….…….…………………….……………………....Page 15**

**Supplementary Figure S8 ….…….…………………….……………………....Page 16**

**Supplementary Figure S9 ….…….…………………….……………………....Page 17**

**Supplementary Figure S10 …...….…………………….……………………....Page 18**

**Supplementary Figure S11 …...….…………………….……………………....Page 19**

**Supplementary Table S1.** The results of the gene expression analysis as determined by the paired-end transcriptome sequences

| **gene** | **Ch** | **NHEKs** | **A431** | **Fold-change** | **P value** | **Q value** |
| --- | --- | --- | --- | --- | --- | --- |
| *ALPPL2* | 2 | 0.0068506 | 23.2066 | 3387.5281 | 4.32E-05 | 0.00687406 |
| *MPPED2* | 11 | 0.0104194 | 23.0187 | 2209.215502 | 8.89E-08 | 6.25E-05 |
| *ABO* | 9 | 0.011938 | 25.4593 | 2132.626906 | 4.66E-05 | 0.0071325 |
| *MAGEA4* | X | 0.125205 | 239.365 | 1911.784673 | 1.58E-07 | 9.21E-05 |
| *PSCA* | 8 | 0.0617987 | 78.5782 | 1271.518657 | 1.94E-08 | 1.48E-05 |
| *CDH5* | 16 | 0.0141485 | 13.2522 | 936.6505283 | 7.99E-09 | 7.48E-06 |
| *ALPP* | 2 | 0.0401729 | 36.1771 | 900.5349377 | 1.63E-09 | 2.11E-06 |
| *PPARGC1A* | 4 | 0.0182892 | 11.1838 | 611.4974958 | 5.51E-07 | 0.000211357 |
| *GPRC5B* | 16 | 0.0165796 | 9.51617 | 573.968612 | 1.51E-05 | 0.00339297 |
| *NUP210* | 3 | 0.018123 | 9.93512 | 548.2050433 | 2.07E-08 | 1.52E-05 |
| *AIM2* | 1 | 0.0392014 | 18.7426 | 478.1104756 | 3.96E-05 | 0.00660649 |
| *B3GALT5* | 21 | 0.00980626 | 4.19891 | 428.1866889 | 0.0003125 | 0.031555 |
| *ALDH1A1* | 9 | 0.224321 | 84.4143 | 376.3102875 | 1.26E-08 | 1.01E-05 |
| *HS6ST2* | X | 0.0162596 | 5.99883 | 368.9408104 | 0.000464262 | 0.0414225 |
| *SPP1* | 4 | 0.261496 | 95.5019 | 365.213617 | 2.43E-07 | 0.000120529 |
| *MMP13* | 11 | 0.068094 | 23.5192 | 345.393133 | 1.04E-07 | 6.75E-05 |
| *AMOT* | X | 8.66812 | 0.0319667 | 0.003687847 | 1.80E-05 | 0.00362679 |
| *FAM171A1* | 10 | 8.31282 | 0.0293358 | 0.003528983 | 2.32E-05 | 0.00438732 |
| *KRTDAP* | 19 | 130.228 | 0.453588 | 0.00348303 | 7.74E-06 | 0.00197689 |
| *INPP5D* | 2 | 20.0091 | 0.068223 | 0.003409599 | 9.96E-08 | 6.72E-05 |
| *CCDC147* | 10 | 8.31706 | 0.0273522 | 0.003288686 | 0.000129506 | 0.015599 |
| *KLK5* | 19 | 200.312 | 0.631658 | 0.003153371 | 2.09E-05 | 0.00401163 |
| *NOS1* | 12 | 2.25625 | 0.0068202 | 0.003022803 | 4.57E-05 | 0.00707434 |
| *KANK4* | 1 | 8.72754 | 0.025726 | 0.002947681 | 4.12E-06 | 0.00115736 |
| *SCN4B* | 11 | 8.70457 | 0.0252213 | 0.002897478 | 5.32E-05 | 0.00773505 |
| *IPW,SNORD116-21* | 15 | 7.02215 | 0.0194449 | 0.002769081 | 1.81E-05 | 0.00362679 |
| *L1CAM* | X | 53.6852 | 0.147471 | 0.002746958 | 3.36E-06 | 0.00101144 |
| *MCAM* | 11 | 27.3559 | 0.0724659 | 0.002649004 | 3.85E-06 | 0.00110049 |
| *ANPEP* | 15 | 18.363 | 0.0439025 | 0.002390813 | 3.82E-06 | 0.00110049 |
| *ODZ4* | 11 | 1.65746 | 0.00364939 | 0.002201797 | 0.000257214 | 0.0269404 |
| *SDK2* | 17 | 4.45279 | 0.0091555 | 0.002056127 | 5.07E-06 | 0.00140243 |
| *SPG20* | 13 | 12.731 | 0.0260093 | 0.00204299 | 0.000183619 | 0.0210637 |
| *ZNF528* | 19 | 6.03189 | 0.0118718 | 0.001968172 | 0.000229103 | 0.0247651 |
| *COL5A3* | 19 | 3.76942 | 0.00733164 | 0.001945031 | 0.000254308 | 0.0268215 |
| *SLC6A15* | 12 | 21.4863 | 0.0410586 | 0.00191092 | 2.28E-06 | 0.00073422 |
| *THBS2* | 6 | 171.094 | 0.32279 | 0.001886624 | 0.000151707 | 0.0177656 |
| *SFRP1* | 8 | 30.4994 | 0.0575241 | 0.001886073 | 5.07E-09 | 5.29E-06 |
| *SLC35F3* | 1 | 10.3018 | 0.0184724 | 0.001793124 | 1.30E-06 | 0.000456842 |
| *MAP1B* | 5 | 5.55239 | 0.00971595 | 0.001749868 | 9.68E-09 | 8.59E-06 |
| *PLD5* | 1 | 11.8741 | 0.0183418 | 0.00154469 | 7.53E-05 | 0.0103246 |
| *POSTN* | 13 | 19.1432 | 0.0290157 | 0.001515718 | 2.09E-05 | 0.00401162 |
| *SPARC* | 5 | 157.798 | 0.225471 | 0.001428858 | 1.96E-07 | 0.000106805 |
| *DSC1* | 18 | 21.8608 | 0.0312242 | 0.001428319 | 1.05E-06 | 0.000386376 |
| *COL1A2* | 7 | 39.5277 | 0.0540016 | 0.001366171 | 4.29E-07 | 0.000180883 |
| *CRYAB* | 11 | 185.087 | 0.248664 | 0.001343498 | 5.15E-07 | 0.000203406 |
| *XG* | X | 59.3771 | 0.0736853 | 0.001240972 | 1.17E-07 | 7.29E-05 |
| *DYSF* | 2 | 11.7121 | 0.0136544 | 0.001165837 | 8.98E-05 | 0.0114776 |
| *PSG4* | 19 | 29.5124 | 0.030592 | 0.001036581 | 1.90E-05 | 0.00377267 |
| *AUTS2* | 7 | 9.17453 | 0.0094456 | 0.001029546 | 5.90E-05 | 0.00849743 |
| *IGFBP5* | 2 | 7.73029 | 0.00777183 | 0.001005374 | 1.06E-08 | 8.92E-06 |
| *LGALS7* | 19 | 1295.99 | 1.28328 | 0.000990193 | 1.09E-10 | 2.30E-07 |
| *KRT1* | 12 | 200.904 | 0.185902 | 0.000925328 | 4.50E-11 | 1.08E-07 |
| *C14orf34* | 14 | 54.9006 | 0.0506997 | 0.000923482 | 3.15E-05 | 0.0055983 |
| *MGC16121,MIR424,MIR503* | X | 32.6174 | 0.0287257 | 0.000880686 | 0.000376488 | 0.0360723 |
| *FBN2* | 5 | 18.6295 | 0.0149198 | 0.00080087 | 4.51E-05 | 0.00704115 |
| *MGST1* | 12 | 207.59 | 0.153643 | 0.000740127 | 6.96E-06 | 0.001806 |
| *CCND2* | 12 | 76.5305 | 0.0560786 | 0.000732761 | 1.76E-11 | 4.96E-08 |
| *GJA1* | 6 | 224.693 | 0.160729 | 0.000715327 | 1.43E-11 | 4.96E-08 |
| *KRT75* | 12 | 169.152 | 0.116323 | 0.000687683 | 1.63E-11 | 4.96E-08 |
| *PLXDC2* | 10 | 7.05273 | 0.00432277 | 0.000612922 | 3.33E-07 | 0.000144035 |
| *CXCL14* | 5 | 622.139 | 0.368418 | 0.00059218 | 7.57E-12 | 4.96E-08 |
| *EPGN* | 4 | 143.547 | 0.070129 | 0.000488544 | 4.22E-05 | 0.00684248 |
| *SESN3* | 11 | 22.9844 | 0.01113 | 0.000484241 | 4.22E-10 | 6.42E-07 |
| *A2ML1* | 12 | 87.0997 | 0.0368378 | 0.000422938 | 2.13E-09 | 2.57E-06 |
| *MFAP5* | 12 | 124.895 | 0.0504743 | 0.000404134 | 4.57E-10 | 6.42E-07 |
| *SLC2A3* | 12 | 45.8989 | 0.0147272 | 0.000320862 | 1.67E-11 | 4.96E-08 |
| *DSG1* | 18 | 50.9796 | 0.0141285 | 0.00027714 | 1.58E-10 | 2.96E-07 |
| *LGALS7B* | 19 | 1941.07 | 0.516477 | 0.000266079 | 4.64E-13 | 7.83E-09 |
| *CDKN2B* | 9 | 52.3773 | 0.0128745 | 0.000245803 | 4.77E-07 | 0.000195983 |
| *IGFL3* | 19 | 216.543 | 0.0211611 | 9.77224E-05 | 3.64E-10 | 6.13E-07 |

The differential expression between the samples was analyzed with Cuffdiff software program by calculating the fragments per kilobase per million map reads (FPKM) and by testing the statistical significance of the differences. The fold-changes of the 70 genes that were significantly up- or down-regulated (FDR<0.01, >2^8^-fold difference) in A431 in comparison to normal human epidermal keratinocytes (NHEKs) are shown. Ch, chromosome.

**Supplementary Table S2.** The putative point mutations in A431

| **Gene** | **Ch** | **Position** | **Reference** | **Alternatives** | **Type** | **Function** | **Homo/hetero** | **Amino acid change** |
| --- | --- | --- | --- | --- | --- | --- | --- | --- |
| *FBXW7* | 4 | 153249393 | G | T/T | SNV | Missense | homo | S→Y |
| *FAT1* | 4 | 187525020 | A | C/C | SNV | Missense | homo | S→A |
| *FAT1* | 4 | 187539588 | T | C/C | SNV | Missense | homo | I→V |
| *FAT1* | 4 | 187540260 | G | A/A | SNV | Nonsense | homo | Q→* |
| *FAT1* | 4 | 187629538 | C | T/T | SNV | Missense | homo | V→I |
| *FAT1* | 4 | 187629770 | A | C/C | SNV | Missense | homo | S→R |
| *KLLN,PTEN* | 10 | 89623716 | G | G/A | SNV | Missense | hetero | G→R |
| *BRCA2* | 13 | 32972753 | C | C/A | SNV | Missense | hetero | S→Y |
| *RB1* | 13 | 49054198 | AG | AG/- | DEL | Frameshift | hetero | E→ |
| *KNSTRN* | 15 | 40675155 | C | C/A | SNV | Missense | hetero | A→E |
| *TP53* | 17 | 7577120 | C | T/T | SNV | Missense | homo | R→H |
| *NOTCH3* | 19 | 15281200 | C | C/A | SNV | Missense | hetero | V→L |
| *NOTCH3* | 19 | 15296484 | A | A/C | SNV | Missense | hetero | L→R |

The putative point mutations were detected using Samtools (v1.0) software program. Ch, chromosome; SNV, single nucleotide variation; DEL, deletion variation.

No nucleotide changes were found in *BRAF, CARD11, CCND1, CDK4, CDKN2A, CREBBP, EGFR, EP300, EZH2, FGFR3, HDAC9, HRAS, HSPB2, KRAS, KRT1, KRT5, KRT10, KRT14, MDM2, MAP2K1, MAP2K2, MYC, NFKB1, NFKB2, NOTCH1, NOTCH2, NOTCH4, NRAS, PIK3CA, PI3CG, RAC1, SIRT1, SMAD4, TERT,* or *WT1*.

**Supplementary Table S3.** The fusion gene candidates identified by the transcriptome analysis

The putative gene fusion transcripts were detected in A431 using deFuse (v0.61) and FusionHunter (v1.4) software programs. NHEKs, normal human epidermal keratinocytes; BCC, basal cell carcinoma.

|  | **5’ Fusion partner** | | **3’ Fusion partner** | |  | | | | | |
| --- | --- | --- | --- | --- | --- | --- | --- | --- | --- | --- |
|  | **Gene name** | **Ch** | **Gene name** | **Ch** | **A431** | **DJM-1** | **NHEKs** | **Melanoma** | **BCC** | **Normal skin** |
| A431 | *CLN6* | 15 | *CALML4* | 15 | - | - | - |  |  |  |
|  | *EGFR* | 7 | *PPARGC1A* | 4 | ＋ | - | - | 0/7 | 0/5 | 0/8 |
|  | *MTATP6* | MT | *MTND1* | MT | - | - | - |  |  |  |
|  | *RMND5A* | 2 | *ANAPC1* | 2 | - | + | + |  |  |  |

**Supplementary Table S4.** The sensitivity and specificity of *EGFR-PPARGC1A* for cSCC

|  | cSCC (n=) | Other tumors (n=) | Total (n=) |
| --- | --- | --- | --- |
| Fusion (+) | 31 | 0 | 31 |
| Fusion (–) | 71 | 106 | 177 |
| Total | 102 | 106 | 208 |

**Supplementary Fig.S1: The differential expression between A431 and NHEKs according to the transcriptome analysis**


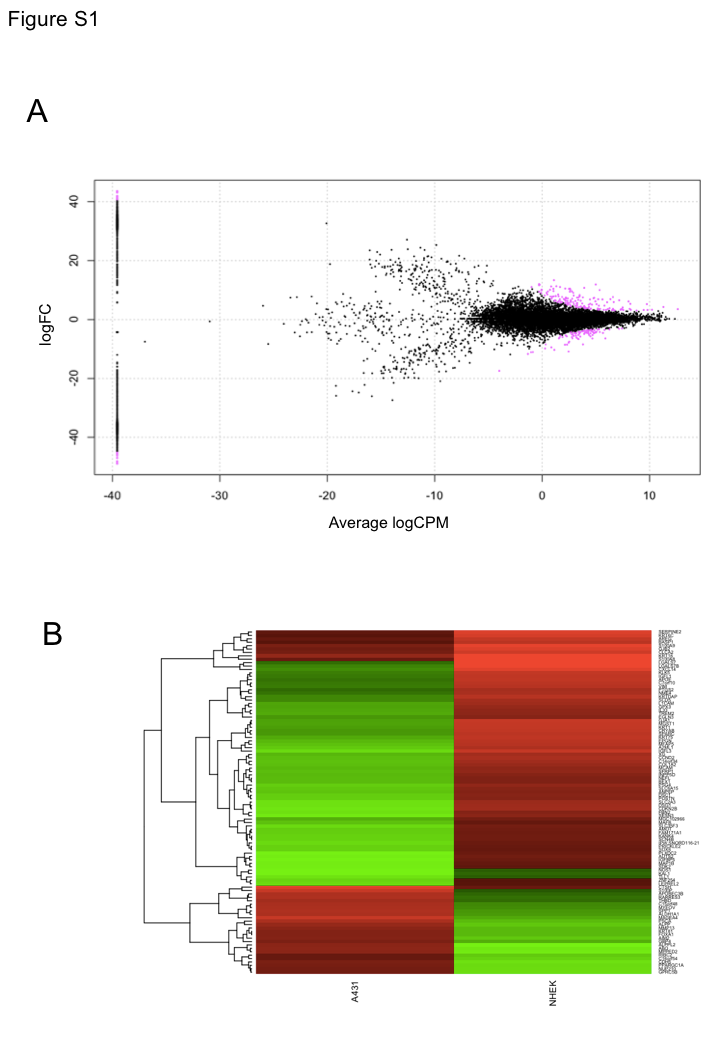


A: An MA-plot for differential expression by transcriptome analysis with NHEKs and A431, annotated to illustrate the use of the grammar of graphics. The x axis indicates the average log2-counts-per-million (logCPM), while the y axis shows the log2 fold-change (logFC). Differently expressed genes (DEGs) (FDR<0.01) are shown in red.

B: A hierarchical clustering heatmap for the genes that were differently expressed between A431 and NHEKs. A heatmap of the 101 differentially expressed genes (FDR<0.01) is shown. The genes that were up- or down-regulated in A431 are indicated by red or green, respectively.

**Supplementary Fig.S2: A schematic representation of the fusion genes of cSCC**


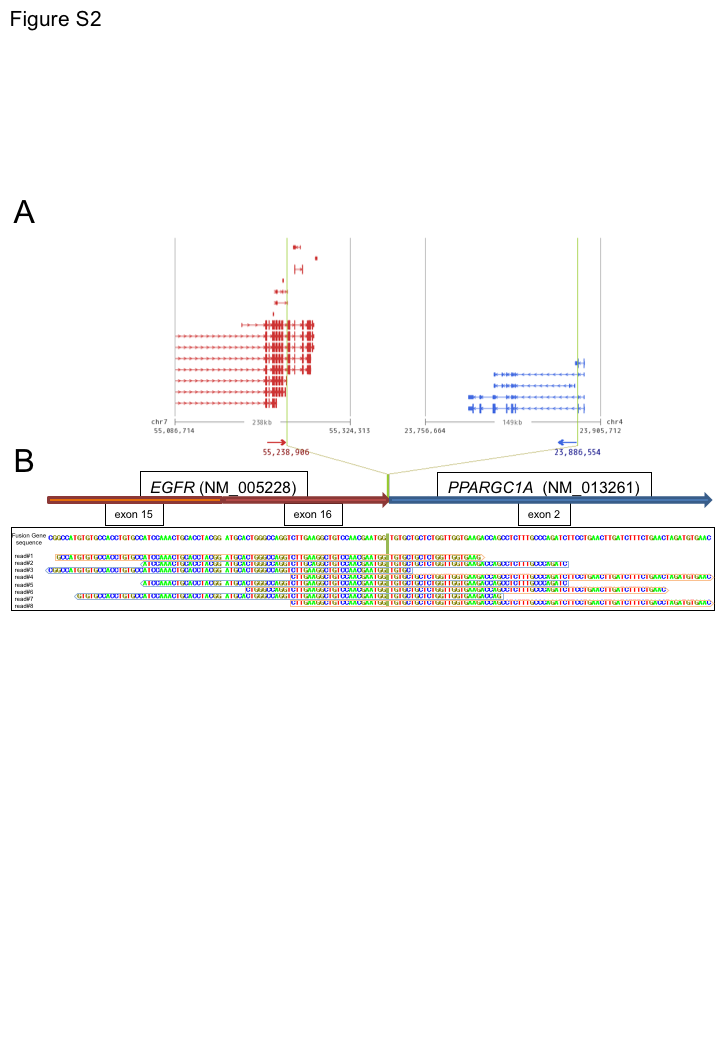
A: A schematic representation of the genomic rearrangement from A431 harboring the *EGFR-PPARGC1A* fusion oncogene: chr7(+):55,238,906|chr4(-):23,886,554.

B: *EGFR-PPARGC1A* fusion in A431 confirmed by the transcriptome sequences.

**Supplementary Fig.S3: A schematic representation of EGFR-PPARGC1A fusion protein**


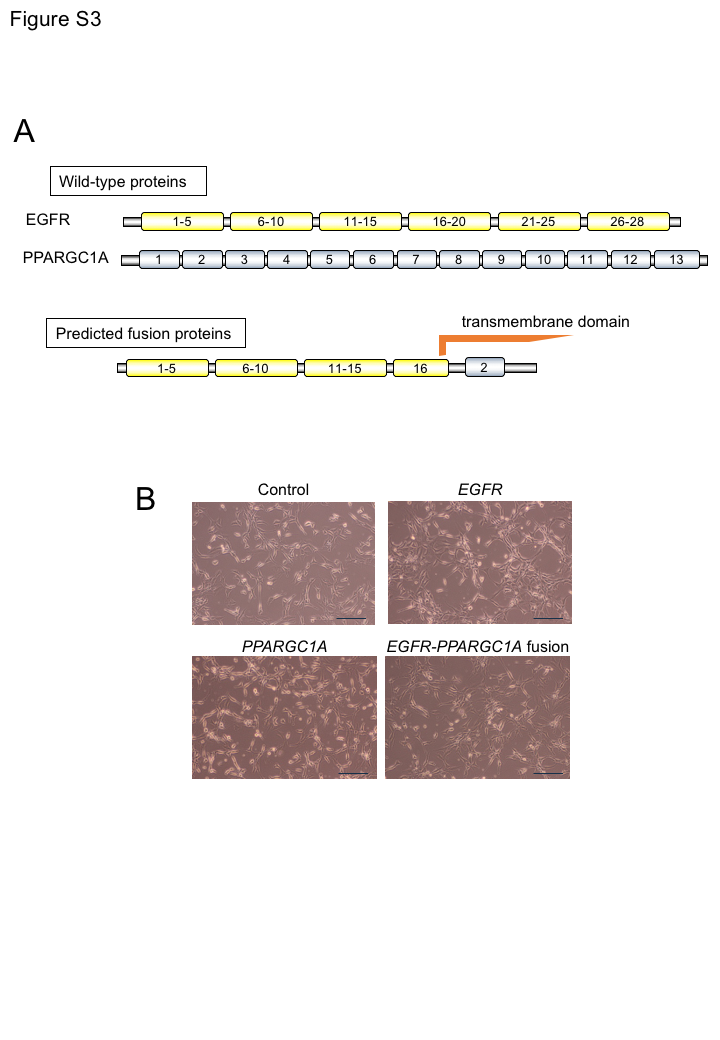
A: A schematic representation of the wild-type full-length EGFR protein, full-length PPARGC1A protein, and the predicted EGFR-PPARGC1A fusion protein product identified in this study. The numbers indicate the exon numbers. The extracellular domain of EGFR consists of 1-15 exons, and the transmembrane domain starts from exon16 according to NCBI database.

B: The microscopic characteristics of NIH3T3 transfected with the control vector, full-length wild-type *EGFR*, full-length *PPARGC1A*, or the *EGFR-PPARGC1A* fusion gene. Bar=200μm.

**Supplementary Fig.S4: The effects of *EGFR* blockade on A431**

**
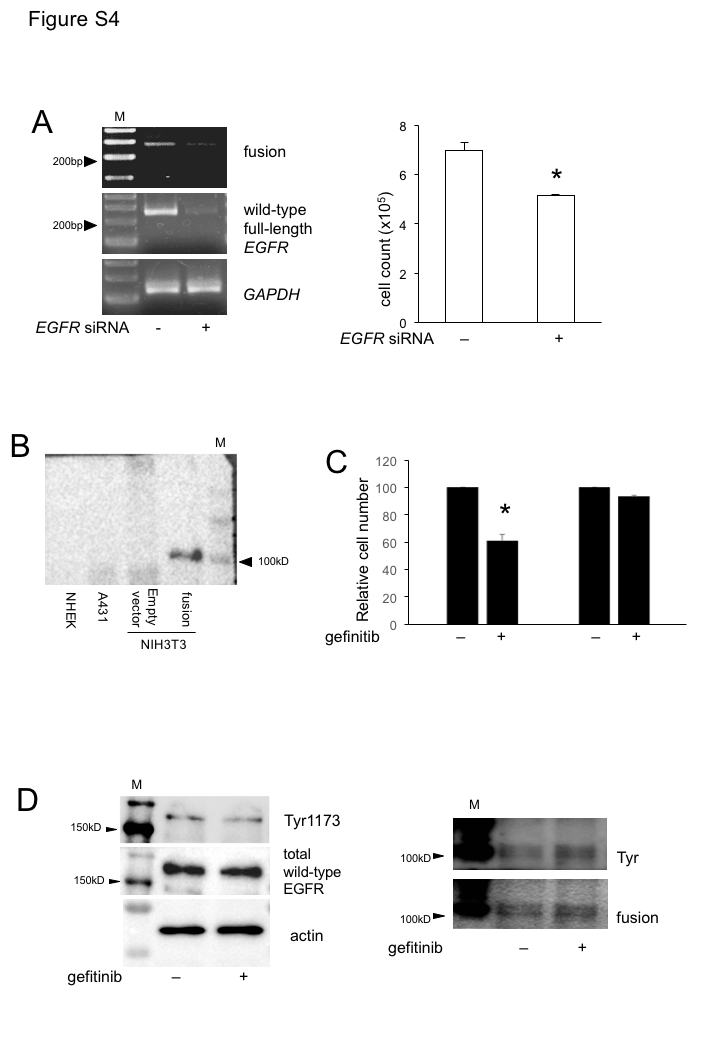
**

A: A431 were transfected with control or *EGFR* siRNA. (Left) To show the transfection efficiency of *EGFR* siRNA, the PCR products obtained using the *EGFR-PPARGC1A*- or wildtype *EGFR*-specific primer pair were run out on agarose gels containing ethidium bromide. The *GAPDH* levels were shown as the control. M, 100-bp ladder. (Right) The cells were counted 72 hours after the transfection, as described in Fig.3D. *P<0.05 (n=3).

B: Cell lysates were immunoprecipitated with antibody against His-tag, followed by immunoblotting with EGFR extracellular domain antibody. M, molecular marker. Cropped images were displayed and original blots are shown in the Supplementary Fig.S10.

C: A431 and DJM-1 were cultured in the presence of vehicle or gefitinib (0.1μM). The cells were counted after 72 hours as described in Fig.3D. *P<0.05 (n=3).

D: Cells were treated with vehicle or gefitinib for 3 hours. (Left) Immunoblots of cell lysates were performed with antibodies against phospho-EGFR Tyr1173 and total wild-type full-length EGFR. Actin was shown as the loading control. M, molecular marker. (Right) Cell lysates were immunoprecipitated with antibody to the extracellular domain of EGFR, followed by immunoblotting with anti-phosphotyrosine (4G10) antibody (Tyr). The same membrane was then stripped and reprobed with anti-EGFR extracellular domain antibody to determine the abundance of total EGFR-PPARGC1A fusion protein. M, molecular marker. Cropped images were displayed and original blots are shown in the Supplementary Fig.S11.

**Supplementary Fig.S5: Original blot for Fig.4A**


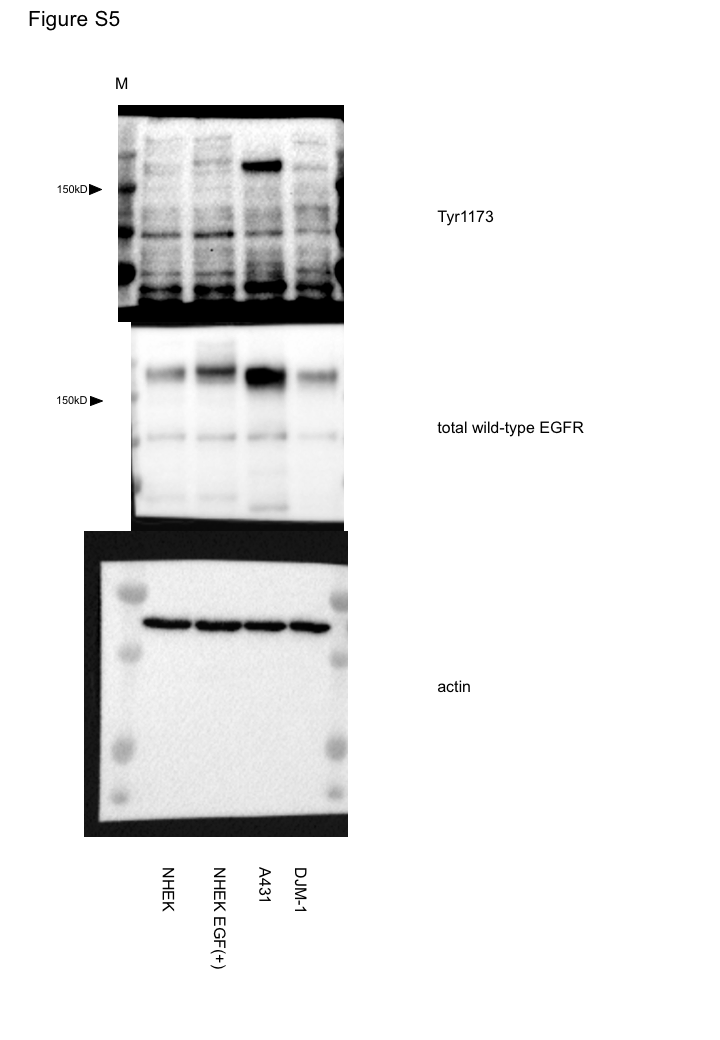


**Supplementary Fig.S6: Original blot for Fig.4B**


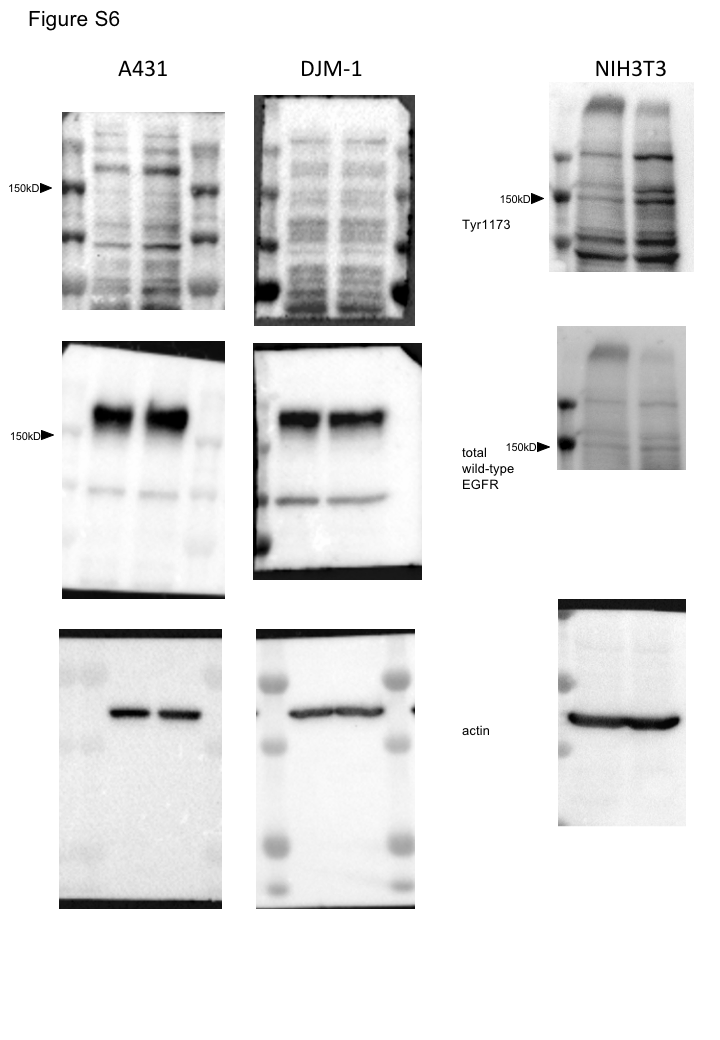


**Supplementary Fig.S7: Original blot for Fig.4C**


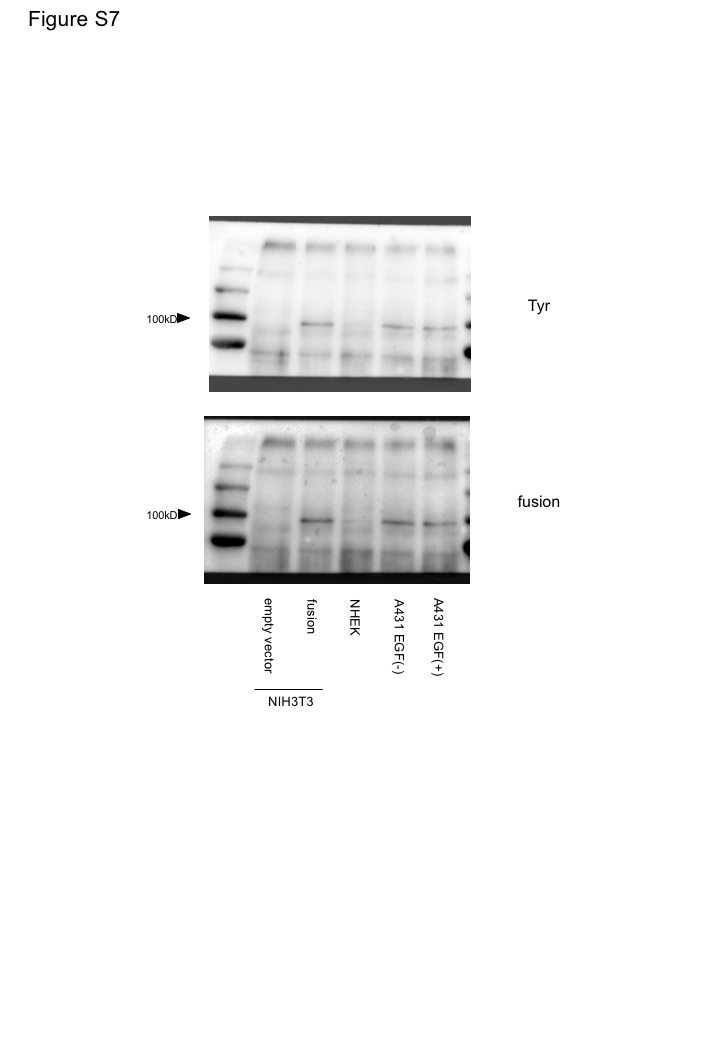


**Supplementary Fig.S8: Original blot for Fig.4D**


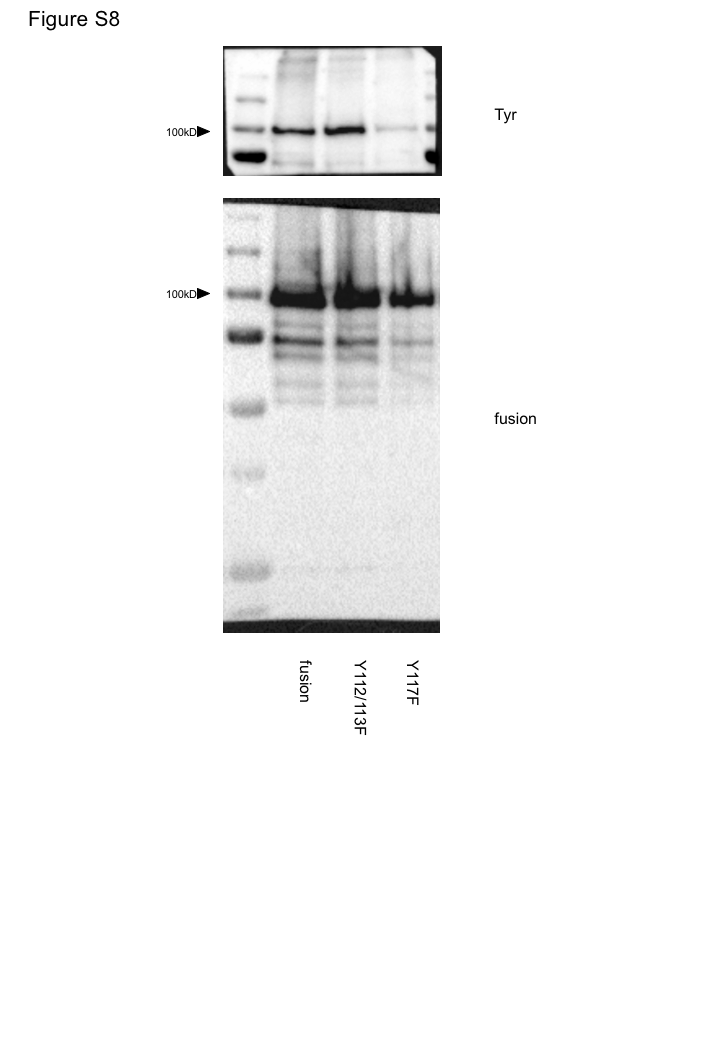


**Supplementary Fig.S9: Original blot for Fig.4E**


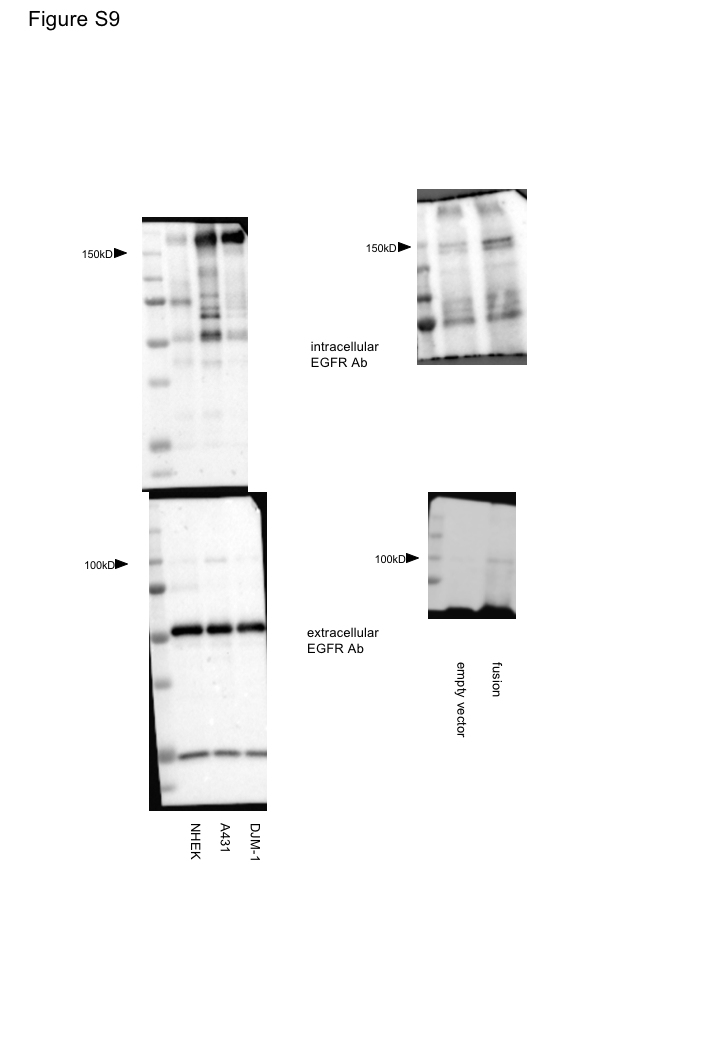


**Supplementary Fig.S10: Original blot for Supplementary Fig.S4B**


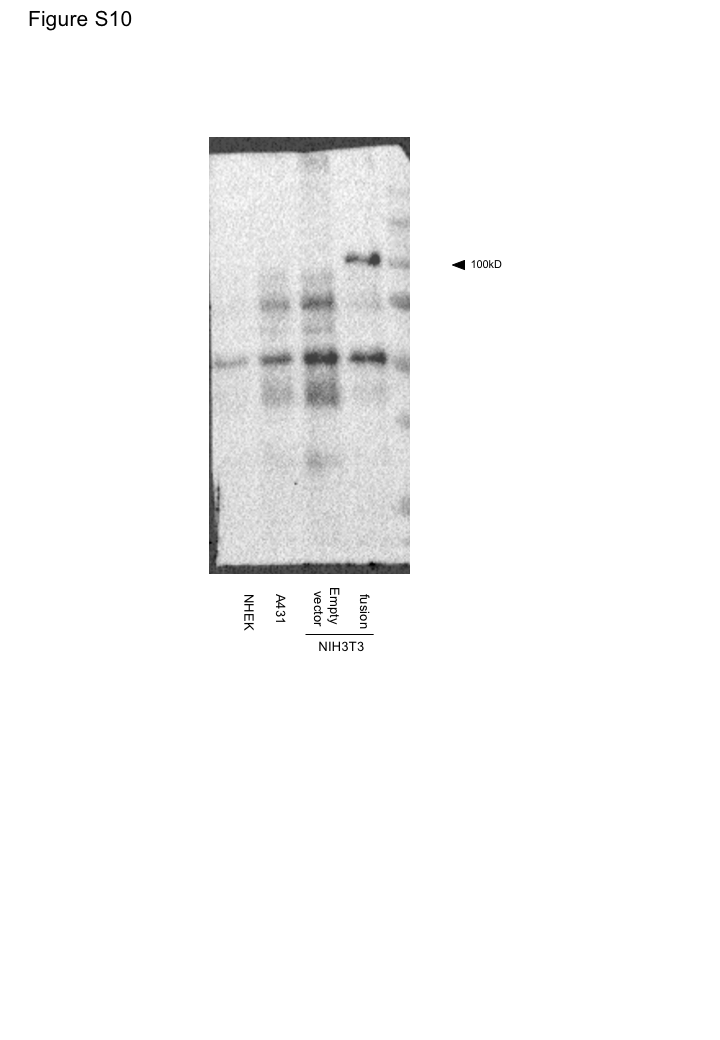


**Supplementary Fig.S11: Original blot for Supplementary Fig.S4D**


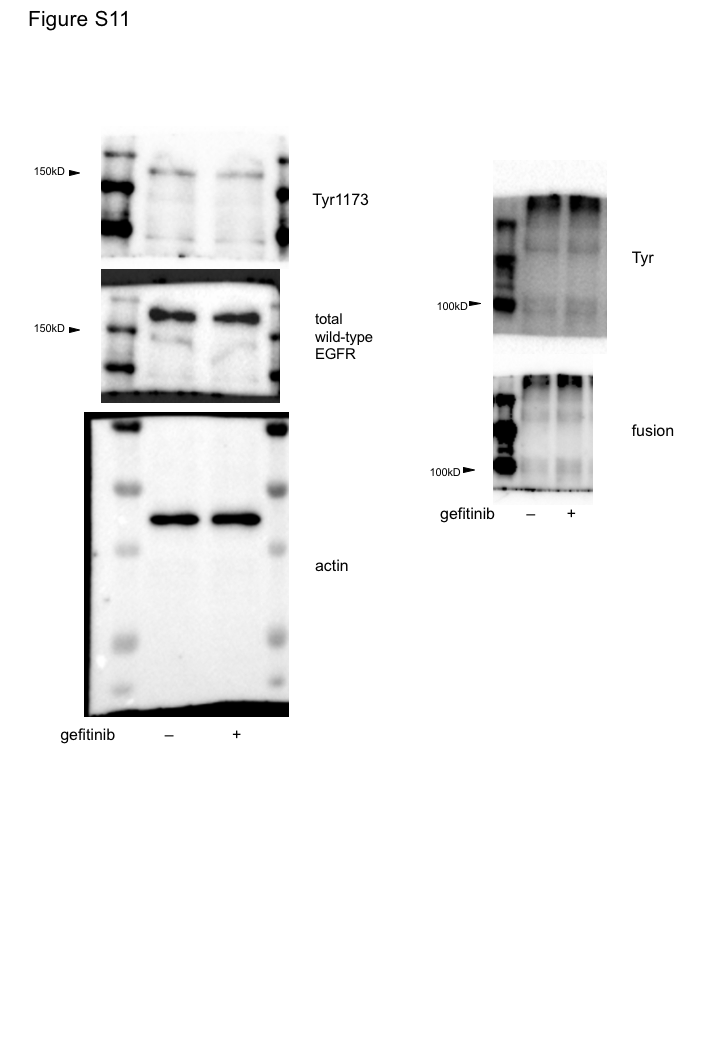

Supplement: Supplementary file 1 — Supplementary Data [file 41598_2017_12836_MOESM1_ESM.doc]
